# Comparative analysis of nanosilver toxicity in *C. elegans*: influence of exposure media on accumulation, physiological and biochemical effects

**DOI:** 10.1007/s11356-025-37339-7

**Published:** 2026-01-22

**Authors:** Andy Joel Taipe Huisa, Marcelo Estrella Josende, Vivien Michaelis, Ann-Kathrin Weishaupt, Anna Gremme, Lucie M. Lindenbeck, Adam Slabon, Christian W. Lehmann, Gürbüz Dursun, Merve Seckin Altuncu, Guillaume Delaittre, José M. Monserrat, Julia Bornhorst

**Affiliations:** 1 Physiological Sciences Post Graduation Program, Institute of Biological Sciences (ICB), Federal University of Rio Grande - FURG, Rio Grande, RS Brazil; 2 Food Chemistry With Focus on Toxicology, Faculty of Mathematics and Natural Sciences, University of Wuppertal, Gaußstr. 20, 42119 Wuppertal, Germany; 3 TraceAge-DFG Research Unit on Interactions of Essential Trace Elements in Healthy and Diseased Elderly (FOR 2558, Berlin-Potsdam-Jena-Wuppertal, 14558 Nuthetal, Germany; 4 Inorganic Chemistry, Faculty of Mathematics and Natural Sciences, University of Wuppertal, Gaußstr. 20, 42119 Wuppertal, Germany; 5 Max-Planck-Institut Für Kohlenforschung, Chemische Kristallographie Und Elektronenmikroskopie, 45470 Mühlheim an Der Ruhr, Germany; 6 Organic Functional Molecules, Organic Chemistry, University of Wuppertal, Gaußstrasse 20, 42119 Wuppertal, Germany

**Keywords:** Stability, Ionic strength, Bioavailability, Oxidative stress, Biofilm, Test media

## Abstract

*Caenorhabditis elegans* is widely used in nanotoxicology studies involving silver nanoparticles (AgNP). However, variations in test media composition can influence AgNP toxicity in worms, complicating cross-study comparisons. This study evaluated the effects of six commonly used media: M9 buffer, K-medium, S-basal, EPA water, Nematode Growth Medium agar (NGM), and “Biofilm” (a combination of NGM and EPA water) on biological responses in worms without the presence of AgNP and the toxicity and accumulation of citrate-coated AgNP (cit-AgNP). Our results indicate that, in the absence of AgNP, NGM and Biofilm were the least disruptive to worm size and DAF-16 translocation (p < 0.05), suggesting lower systemic stress responses. In contrast, all liquid media significantly affected at least one of these parameters. In the presence of cit-AgNP, toxicity patterns in *C. elegans* at 5 mg/L (administered in 1 mL of food source) varied across the tested media (EPA water, NGM, and Biofilm). Worms in EPA water exhibited the most pronounced effects on size, brood size, and GSH/GSSG levels, whereas those in NGM agar were unaffected. These differences are attributed to the media's impact on worms, observed for EPA water, and reduced Ag accumulation in worms due to increased Ag migration to agar, particularly in NGM. This study demonstrates that the treatment scenarios currently used for assessing AgNP toxicity in *C. elegans* produce varying outcomes due to the media’s intrinsic effects on worms and differences in Ag accumulation. Our findings underscore the need for standardized media to ensure accurate and comparable toxicity assessments.

**Supplementary Information:**

The online version contains supplementary material available at 10.1007/s11356-025-37339-7.

## Introduction

Silver nanoparticles (AgNP) have been extensively studied for their antimicrobial properties (Bruna et al. 2021); these applications have led to increased production, which is estimated to exceed 600 tons per year, and consequently to greater release into the environment. This can occur both through the disposal of products containing these nanoparticles and through the washing of textiles that incorporate them. As a result, environmental concentrations of AgNP could reach up to 10 mg/L, leading to higher exposure of non-target organisms and potential toxicity (Yang et al. 2024). However, their toxicity might be challenging to evaluate due to factors such as size, morphology, coating, surface charge, and interaction with biological systems (Contreras et al. 2014; Kim et al. 2012; Moon et al. 2019; Schultz et al. 2020). *Caenorhabditis elegans* is a widely used model in nanotoxicology due to its ease of maintenance, transparent body, high reproductive rate, short life cycle, and availability of numerous transgenic strains (Brenner 1974; Gonzalez-Moragas et al. 2015). AgNP exposure in *C. elegans* can impact growth, reproduction, induce oxidative stress, genetic damage, and cause multigenerational epigenetic effects (Contreras et al. 2014; Hunt et al. 2013; Taipe Huisa et al. 2024; Wamucho et al. 2020). However, comparisons of AgNP toxicity between studies in worms might be complicated due to the wide variety of exposure media available that are currently used, such as moderately hard reconstituted water (MHRW or EPA water), Nematode Growth Medium (NGM), Simulated Soil Pore Water (SSPW), M9 buffer, K-medium, S-basal, Biofilm (NGM + EPA water), and others (Table S1, Figure S1). These media present different compositions and consequently different ionic strengths (mainly due to different chloride, sodium, and potassium ion concentrations), which can alter AgNP stability and cause nanoparticle agglomeration, especially in media with high ionic strength such as K-medium, S-basal, and M9 buffer (Table S1) (Yang et al. 2018, 2019). In addition, it has been observed that the ionic strength of the media can influence the bioavailability of AgNP and consequently their toxicity in worms (Yang et al. 2018, 2019). Some efforts have been made to address this issue, such as the introduction of the aforementioned EPA water and SSPW in 2013, both having a low ionic strength and consequently reducing AgNP agglomeration (NANoREG 2016; Tyne et al. 2013). Despite this, the use of different media persists (Figure S2), and the ISO 10872 guideline for *C. elegans* toxicity testing does not consider nanomaterials, further complicating comparisons (ISO 10872:^2020^). Furthermore, approximately only 15% of the studies have quantified Ag in worms (Yang et al. 2014; Hunt et al. 2013; Contreras et al. 2014; Maurer et al. 2016; Luo et al. 2016; Kleiven et al. 2018; Yang et al. 2018; Nie et al. 2020; Majumder et al. 2022; Luo et al. 2022; Mello et al. 2022), leading to challenges in interpreting the results due to the potential variability in bioavailability caused by using different media (Table S2). Consequently, although the concentrations used in these studies might be the same, the concentration of AgNP internalized by the worms could differ. Few studies have explored media influence on AgNP toxicity, but they have focused mainly on the impact of media ionic strength on AgNP stability (Luo et al. 2017; Yang et al. 2018, 2019). However, the literature lacks a clear understanding of how the media alone might affect worm health and its influence on AgNP-induced toxicity, especially during the early larval stages when worms are more susceptible to environmental toxicants (Wang et al. 2020).

Therefore, this study aimed to evaluate physiological and biochemical effects of commonly used media (M9 buffer, K-medium, S-basal, EPA water, Nematode Growth Medium (NGM), and “Biofilm” (NGM + EPA water) alone, and their influence on toxicity and accumulation of citrate-coated AgNP (cit-AgNP) in *C. elegans*. We also included a relatively novel medium combining NGM and EPA water referred to as “Biofilm” which consists of a regular NGM plate with a thin layer of EPA water placed on the surface (Josende et al. 2019). Although SSPW is listed as low ionic strength media, it was excluded because its formulation varies across studies, making experimental conditions difficult to replicate for us.

## Materials and methods

### Chemicals

All reagents used in this study were of analytical grade, and ultrapure water (MilliQ-Millipore) was used to dissolve the reagents. The toxicity assays involved the use of silver nanoparticles stabilized with citrate (cit-AgNP) obtained from Sigma Aldrich, Germany. According to the manufacturer, these nanoparticles have an average size of 20 nm, as determined by transmission electron microscopy (TEM). According to our own characterization, they exhibited a characteristic peak at 400 nm due to surface plasmon resonance (SPR) and a particle size of 27.77 ± 0.13 nm (Z-average) and polydispersity index (PDI) of 0.13 ± 0.02 in MilliQ water as determined by dynamic light scattering (DLS) and of 17.54 ± 4.14 nm as measured by TEM, indicating the presence of AgNP (Figure S3).

### Characterization of citrate silver nanoparticles

#### Electron microscopy

Morphological characterization of the nanoparticles was performed using a transmission electron microscope Hitachi H-7500 operated at 100 kV. The sample was prepared by dropping 10 μL of the suspension after 10 min of sonication over a TEM grid (lacey carbon film supported on a copper grid). The particle size and particle size distribution were determined from the TEM measurements of 250 particles.

#### Dynamic light scattering

The particle size and polydispersity index (PDI) of the samples were assessed by dynamic light scattering (DLS) with an Amerigo Particle Size & Zeta Potential Analyzer (Cordouan Technologies, Pessac-Bordeaux, France). All samples were aliquoted into a cuvette and exposed to an external laser of 638 nm with a power of 10%. The assessments were conducted at room temperature at a 170° angle using the Rayleigh scattering model. Data analysis was performed using Amerigo software (v2.3.4.0) and either the Sparse Bayesian Learning (SBL) algorithm or the cumulant algorithm, and the resulting intensity-based hydrodynamic size of the particles (Z-average) was utilized.

### Stability of silver nanoparticles in different media over time

To investigate the impact of different media on cit–AgNP stability over time, we conducted a comparative analysis of four liquid media: EPA water (60 mg/L CaSO₄·2H₂O, 60 mg/L MgSO₄, 4 mg/L KCl, and 96 mg/L NaHCO₃ in MilliQ H_2_O), K-medium (3.0 g/L NaCl and 2.36 g/L KCl in MilliQ H_2_O), M9 buffer (3.0 g/L KH₂PO₄, 6.0 g/L Na₂HPO₄, 5.0 g/L NaCl, 1.0 mL of 1 M MgSO₄ in MilliQ H_2_O), and S-basal (5.85 g/L NaCl, 1.0 g/L K₂HPO₄, 6.0 g/L KH₂PO₄, 1.0 mL of cholesterol solution [5 mg/mL in ethanol] in MilliQ H_2_O). The stability of the nanoparticles was assessed by recording Surface Plasmon Resonance (SPR) in the range of 300–700 nm using a Tecan Infinite Pro M200 spectrophotometer (Tecan, Crailsheim, Germany) over 52 h (duration of all worm experiments). AgNP diluted in ultrapure water was used as a reference. To compare the stability of cit–AgNP in NGM (3 g NaCl, 17 g agar, 2.5 g peptone, 1 mL 1 M CaCl₂, 1 mL 5 mg/mL cholesterol in ethanol, 1 mL 1 M MgSO₄, and 25 mL 1 M KPO₄ buffer per liter of MilliQ H_2_O) and Biofilm media (NGM media + 3 mL EPA water), we conducted a DLS analysis. Briefly, cit–AgNP were placed on NGM and Biofilm plates and allowed to dry. A layer of EPA water was added only to the biofilm medium. After 0 and 52 h, the plates were washed with MilliQ water or EPA water (for NGM and biofilm plates, respectively), and the collected suspension was used for analysis. These experiments were carried out in the absence of bacteria and worms.

### *C. elegans* strains and culture

N2 wild-type and transgenic daf16::GFP (TJ356) strains were obtained from the Caenorhabditis Genetics Center (CGC). Both strains were maintained at 20 °C on peptone rich 8P plates (3 g NaCl, 37.5 g bacteriological agar, and 30 g peptone, supplemented with 1.5 mL of 1 M CaCl₂, 1.5 mL of 1 M MgSO₄, 37.5 mL of 1 M KPO₄ buffer (pH 6.0), and 1.5 mL of cholesterol solution (5 mg/mL) for each 1.5 L of MilliQ H_2_O) (Sangaletti et al. 2013) seeded with *Escherichia coli* NA22 To obtain a synchronized population, gravid adult worms were treated with a bleaching solution (1% NaOCl and 0.5 M NaOH) to allow the release of eggs (Stiernagle 2006) and let them hatch overnight in M9 buffer until they reached the L1 larval stage without the presence of bacteria.

### Exposure design

All experiments were conducted in 10 cm plates containing 5000 L1 worms, either N2 wild-type or transgenic, along with 1 mL of heat-inactivated OP50 *E. coli* (OD_600_ = 1). For exposure to liquid media, in addition to the conditions described above, each plate was filled with 15 mL of liquid media. For the NGM and biofilm groups, plates filled with 25 mL of NGM were used, and bacteria were applied to the surface and allowed to dry for approximately 2 h. Following the drying of the bacteria in the biofilm group, 3 mL of EPA water was added to create a thin layer over the plate surface. All experiments were conducted in the dark. Each experiment was performed in triplicate.

### Effect of the media on worm responses

#### Size

To assess the potential impact of the media alone on the size of worms, exposure was conducted using NGM, Biofilm, and various liquid media: EPA water, K-medium, M9 buffer, and S-basal. Worm size was determined by measuring the length of worms (from head to tail) based on images obtained 4, 30, and 52 h after the initiation of the experiments. Images were captured using a Leica MZ10 F stereomicroscope coupled with a FLEXACAM C1 camera (Leica Microsystems, Germany), and subsequent analysis was performed using the free software ImageJ (NIH). Approximately 30 worms per sample were measured, and the results were expressed as a percentage relative to the NGM group. The experiment was performed in triplicate.

#### DAF-16 nuclear localization assay

For the analysis of DAF-16 translocation, transgenic worms (daf16::GFP) were used. L1 worms were exposed for 52 h in each test medium and then subjected to three washes with 85 mM NaCl (for worms in NGM, K-medium, M9 buffer, and S-basal) or EPA water (for worms in Biofilm and EPA water). Subsequently, the worms were transferred to SuperFrost® microscope slides containing an agarose path (4% agarose) and 10 µL of 5 mM levamisole for immobilization. To verify the localization of DAF-16, a Leica DM6 B Microscope equipped with a Leica DFC7000 T camera (Leica Microsystems, Germany) was used. The experiment was conducted in triplicate, and sample analyses were performed in a blind manner. The results were expressed as the percentage of nuclear DAF-16 localization based on 30 worms per sample. (Gubert et al. 2016).

### Effect of media on silver nanoparticle toxicity and Ag content

Based on AgNP stability and worm fitness results in the media alone, three media were selected for the assessment of AgNP toxicity after 52 h of exposure: EPA water, NGM, and Biofilm. Two concentrations of cit-AgNP – 1 and 5 mg/L – were used for each medium, with the respective control groups being the corresponding media without nanoparticles. The nanoparticles were added to the inactivated OP50 *E. coli* in Milli Q water to achieve 1 and 5 mg/L in the food source, 1 mL of which was subsequently added to each exposure replicate or plate as described in the exposure design (Sect. "Exposure design").

#### Size

After 52 h of exposure, the size of the worms was measured by capturing the images of each sample, as described in Sect. "Size". Approximately 30 worms per sample were measured, and the results were expressed as a percentage relative to the NGM group. The experiment was performed in triplicate.

#### Total brood size

After 52 h of exposure, worms from each plate were transferred to 15 mL falcon tubes and washed three times with 85 mM NaCl (NGM plates) or EPA water (Biofilm and EPA water group). Single worms were then transferred to 3.5 cm plates containing 50 µL of active OP50 *E. coli* and subsequently moved daily to fresh plates until no further eggs were observed. Total brood size was determined by counting the overall number of larvae produced. Additionally, the worms were observed daily for any reproductive abnormalities up to day 10 post exposure. The experiment was performed in triplicate.

#### GSH and GSSG levels

After 52 h of exposure, worms from each plate were transferred to 15 ml falcon tubes and washed three times with 85 mM NaCl (NGM plates) or EPA water (Biofilm and EPA water group), pelletized in 1.5 mL tubes, frozen in liquid nitrogen, and stored at −80 °C. Worm pellets were resuspended in 150 µL cold extraction buffer (16 mM KH_2_PO_4_, 84 mM K_2_HPO_4_, 8.8 mM EDTA, 2 mM NEM, 1% Triton X-100, 0.6% SSA) and samples were subjected to three freeze–thaw in cycles (1 min liquid nitrogen, 1 min 37 °C water bath) followed by homogenizing four times for 20 s using a Bead Ruptor. All extracts were filtered using Spin-X® centrifuge tube filters (0.22 µM; Corning) and centrifuged at 18,620 × g at 4 °C for 5 min. An aliquot was stored at − 20 °C for protein quantification. Quantification of GSH-NEM and GSSG was performed using an Agilent 1290 Infinity II LC System coupled to a Sciex QTrap 6500 + triple quadrupole mass spectrometer with an electrospray ion source in the positive mode (Thiel et al. 2023). The measurement parameters are listed in Table S3. The results are expressed as a percentage of the NGM control. The experiment was performed in triplicate.

#### Ag content in worms

To evaluate the influence of test media on the content of Ag in worms, we quantified the total amount of Ag in worms after 52 h of exposure. To this end, worms from each plate were transferred to 15 mL falcon tubes and washed three times with 85 mM NaCl (NGM plates) or EPA water (Biofilm and EPA water group), and pellets were transferred to 1.5 mL tubes, then frozen in liquid nitrogen and stored at −80 °C. The worm pellets were then subjected to three freeze–thaw cycles (1 min liquid nitrogen, 1 min 37 °C water bath), followed by homogenization by sonication three times for 20 s at 100% amplitude (ultrasonic processor UP100H (100 W, 30 kHz, Hielscher, Germany)). After, the homogenate was centrifugated at 18 000 × g for 5 min at 4 °C and an aliquot of the supernatant was obtained for protein measurement. For Ag measurements, the worm pellets were dried at 95 °C and digested overnight with a 50:50 mixture of 65% HNO_3_ (Suprapur, VWR, Darmstadt, Germany) and 30% hydrogen peroxide (Sigma–Aldrich) at 95 °C. The ashes were diluted in 2% HNO_3_ (1:3) and measured using an inductively coupled plasma-optical emission spectrometer (ICP-OES; Perkin Elmer Avio 220 Max, Germany). The measurement parameters are listed in Table S4**.** ICP measurements were validated using Ag-spiked certified reference material BCR (single cell-protein, Institute for Reference Materials and Measurement of the European Commission, Geel, Belgium), which was digested according to the protocol for worms. The total amount of Ag was normalized to the protein content, which was measured using a NanoDrop One/One^C^ spectrophotometer (Thermo Fisher Scientific, USA). The experiment was performed in triplicate.

#### Ag distribution in exposure system

Although worms can internalize Ag, not all Ag (as Ag ions or AgNP) is expected to be taken up by worms. Therefore, to further investigate the distribution of Ag within the test media system, we quantified the amount of Ag remaining in the system by measuring the Ag in the washing solution after washing the worms, attached to the residual bacteria and/or precipitated agglomerates/aggregates, and incorporated into the agar that resisted removal during the washing steps (specifically for NGM and Biofilm). The separation procedure for each matrix involved the following steps: following plate washing and separation of the worm pellet, all washing solutions containing the remaining bacteria were collected in a 50 mL tube and centrifuged at 2500 × g for 20 min at room temperature. The resulting supernatant (washing solution) was transferred to another tube and stored at −20 °C. The remaining pellet (AgNP attached to bacteria and/or precipitated agglomerates/aggregates) was then transferred to a 1.5 mL tube and dried at 95 °C. Subsequently, the agar from the plates was excised, transferred to a beaker, melted, homogenized, and a 1 mL aliquot was transferred to a 1.5 mL tube and dried at 95 °C. For ICP-OES measurements, the washing solutions were diluted 1:2 in 2% HNO_3_, and the dried samples of the bacterial pellets and agar were digested and diluted in the same manner as the worm pellet samples. The Ag content in the washing media was expressed as µg of Ag per L, while Ag in the bacterial pellet and agar was expressed as ng of Ag per g of wet weight and ng per mL of agar, respectively. The experiment was performed in triplicate.

### Statistical analysis

All results are expressed as mean and standard error. The results obtained with *C. elegans* without AgNP were analyzed using one-way ANOVA, and if AgNP was included, two-way ANOVA was performed (media and AgNP concentration as the two factors). The Newman–Keuls post-hoc test was performed to compare the means of the different groups and, in some cases, with orthogonal contrasts. Previously, normality and variance homogeneity were verified, and mathematical transformations (logarithmic or ranked) were applied if at least one of these two assumptions was violated. In all cases, the significance level (α) was set at 0.05.

## Results and discussion

### Effect of the media alone in worms

**Fig. 1:**
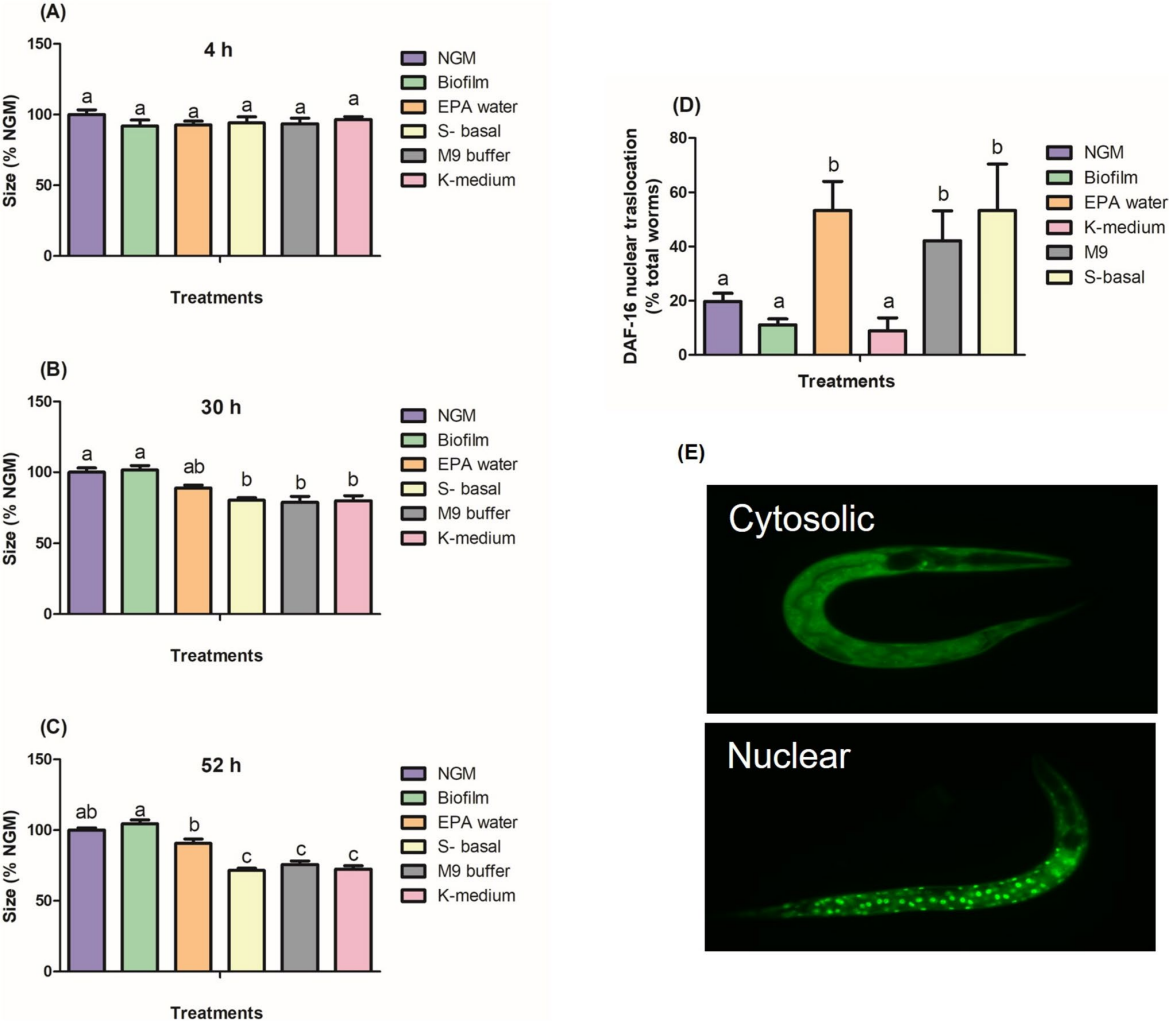
Worm size after 4 h **(A)**, 30 h **(B)**, and 52 h **(C)** of exposure to different media. The results are expressed as a percentage of the NGM group. Nuclear translocation of DAF-16 induced by exposure to different media (**D**). The results are expressed as the percentage of worms that presented nuclear translocation. Thirty worms per sample were counted in three independent blinded experiments. Different lowercase letters indicate significant differences between the treatments by ANOVA (p < 0.05). (**E**) Representative cytosolic and nuclear localizations of DAF-16

**Fig. 2:**
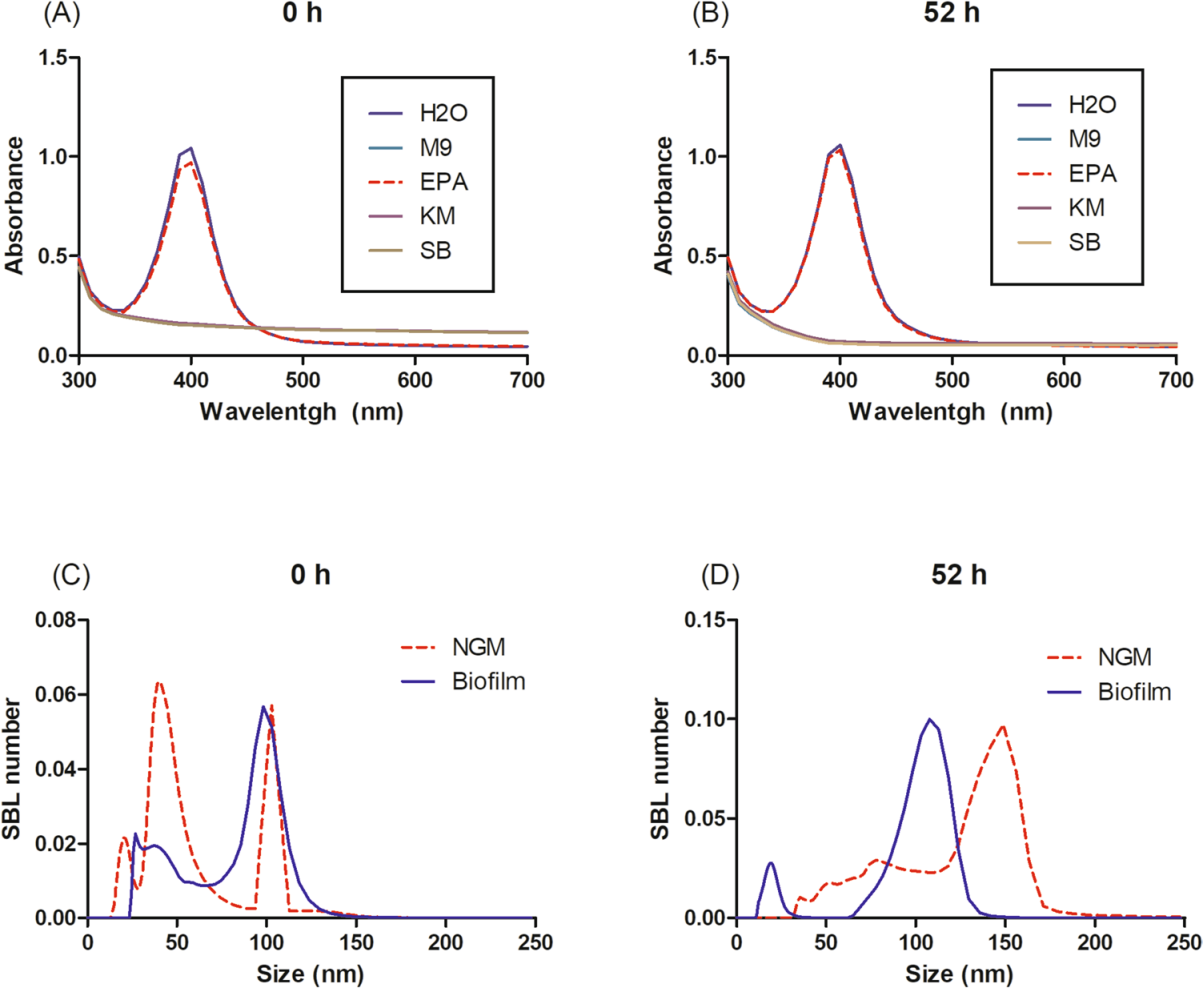
Stability of cit-AgNP in the media. Surface plasmon resonance of cit-AgNP diluted in MilliQ water (H_2_O), M9 buffer (M9), EPA water (EPA), K-medium (KM), and S-basal (SB) after 0 h **(A)** and 52 h (**B**), monitored by UV–VIS spectrophotometry. Effect of NGM and biofilm media on the size distribution of cit-AgNP after 0 h (**C**) and 52 h (**D**) of exposure as measured by DLS. DLS results are expressed as the SBL (Space Bayesian Learning) number based on nine measurements

To evaluate the effect of the media on worm biological responses, we monitored worm size and DAF-16 translocation over 52 h from the L1 stage. At 4 h, no differences in size were observed (Fig. 1A). However, at 30 h, worms in liquid media were significantly smaller than those in NGM and Biofilm (Fig. 1B), a trend that continued at 52 h (Fig. 1C). Among the liquid media, EPA water had the least effect on worm size. The transcription factor DAF-16, a homolog of mammalian forkhead box O (FOXO), regulates, among other, antioxidant genes and translocates to the nucleus under oxidative stress (Henderson and Johnson 2001; Huang et al. 2017) and increased significantly in worms exposed to all liquid media, except K-medium (Fig. 2D). Worms in Biofilm media showed low DAF-16 translocation, similar to that of NGM. In contrast, up to 50% of nuclear DAF-16 localization was observed in worms exposed to EPA water, M9, and S-basal. These results suggest that liquid media hinder worm growth, likely due to the induction of catabolic reactions caused by the increased necessity of ATP consumption induced by constant natatory movement of worms. Also, previous evidence suggests that worms in liquid media may experience delayed development, inhibited growth, altered lipid and protein storage, and upregulation of the daf-16 pathway (Çelen et al. 2018; Houthoofd et al. 2002; Sokolova 2021; Szewczyk et al. 2006). These findings highlight the influence of media on the basal stress response levels, especially in worms exposed to liquid media alone.

### Stability of AgNP in different media over time

When assessing stability via UV–VIS spectrophotometry (Fig. 2A and 2B), cit–AgNP in MilliQ water maintained a consistent peak at 400 nm, indicating well-defined, small nanoparticles over 0 and 52 h. EPA water showed a similar pattern indicating nanoparticle stability over time. In contrast, no peaks were observed in M9 buffer, S-basal, and K-medium at 0 h, suggesting nanoparticle agglomeration. These results are consistent with previous studies with low ionic strength such as EPA water indicating low agglomeration (Yang et al. 2014), while media with high ionic strength such as K-medium showed high agglomeration and aggregation (Meyer et al. 2010; Luo et al. 2017). To compare the effect of NGM and Biofilm media on the retrieved cit–AgNP stability, we performed a dynamic light scattering (DLS) analysis since the UV–VIS technique was not sensitive enough, revealing similar size distribution patterns at 0 h with multimodal peaks around 20, 40, and 100 nm. After 52 h, NGM showed a peak shift to 148 nm, while Biofilm had a smaller shift to 108 nm. Thus, Biofilm media appears to provide better nanoparticle stability than NGM over time (Fig. 2C and 2D).

From these results, we observed that M9 buffer, S-basal, and K-medium greatly affect worms’ fitness and/or have a great influence on the stability of the nanoparticles. Therefore, we proceed to compare the influence of NGM, EPA water, and biofilm media on the toxicity and accumulation of cit-AgNP in worms.

### Effect of media on silver nanoparticle toxicity and accumulation

#### Size and total brood size

**Fig. 3:**
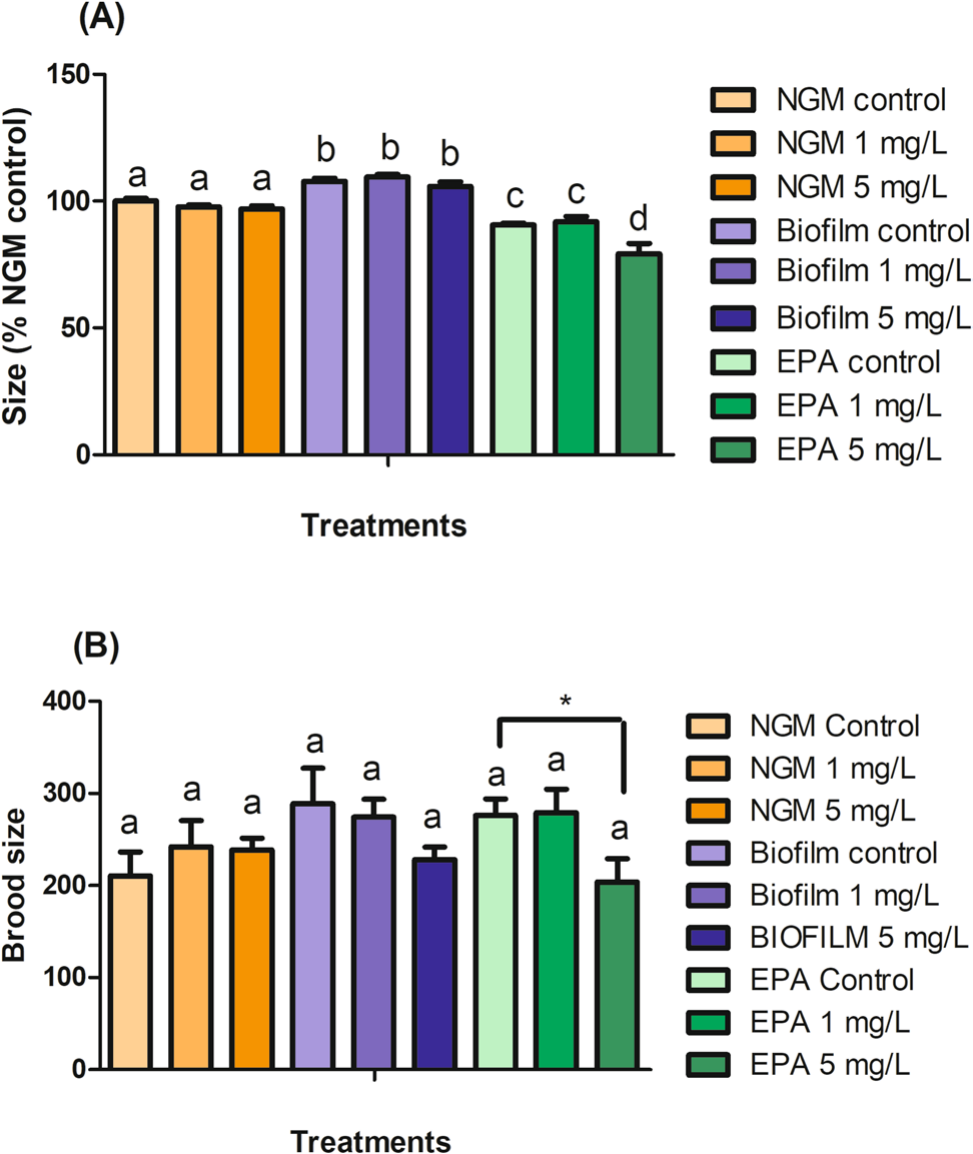
Effects of cit-AgNP on size (**A**) and total brood size (**B**) in worms after 52 h exposure to 1 and 5 mg/L cit-AgNP in NGM, Biofilm, and EPA water**.** The results are expressed as a percentage of the NGM control for size. Different lowercase letters indicate significant differences between the treatments by ANOVA (p < 0.05). Asterisk (*) indicates statistical differences (p < 0.05) after orthogonal contrast

After 52 h, no differences in worm size were observed in the NGM and Biofilm groups compared to the controls, but worm size in the EPA group was significantly reduced at 5 mg/L (Fig. 3A). Notably, the EPA control group showed a reduced size compared to the other controls, indicating a combined effect of the media and AgNP. Also, this could be linked to AgNP agglomeration, which was likely greater in media that included agar, such as NGM and Biofilm, or higher AgNP dissolution in EPA water. This aligns with previous studies, where growth reduction occurred in other liquid media (C. elegans Habitation Reagent and K-medium) at 5 mg/L with 10 nm cit-AgNP (Hunt et al. 2013; Meyer et al. 2010), while higher concentrations were needed in NGM for similar effects (Luo et al. 2017). In terms of reproduction, brood size was significantly reduced at 5 mg/L in EPA water but not in Biofilm and NGM, likely due to pre-existing stress caused by EPA water alone (Fig. 3B). This is consistent with earlier research that found similar brood reductions at 5 mg/L AgNP in EPA water (Yang et al. 2019), while reprotoxicity effects in NGM were observed in concentrations above 10 mg/L (Kim et al. 2012). Additionally, we observed vulvar abnormalities in worms exposed to 5 mg/L AgNP, similar to the effects caused by gold nanoparticles (Moon et al. 2017), although this did not significantly impact reproduction (Figure S4). It is important to note that the studies previously mentioned that were performed in liquid media used AgNP concentrations as the final concentration in the exposure medium, rather than as the concentration in the bacterial volume as ours.

#### GSH and GSSG levels

**Fig. 4:**
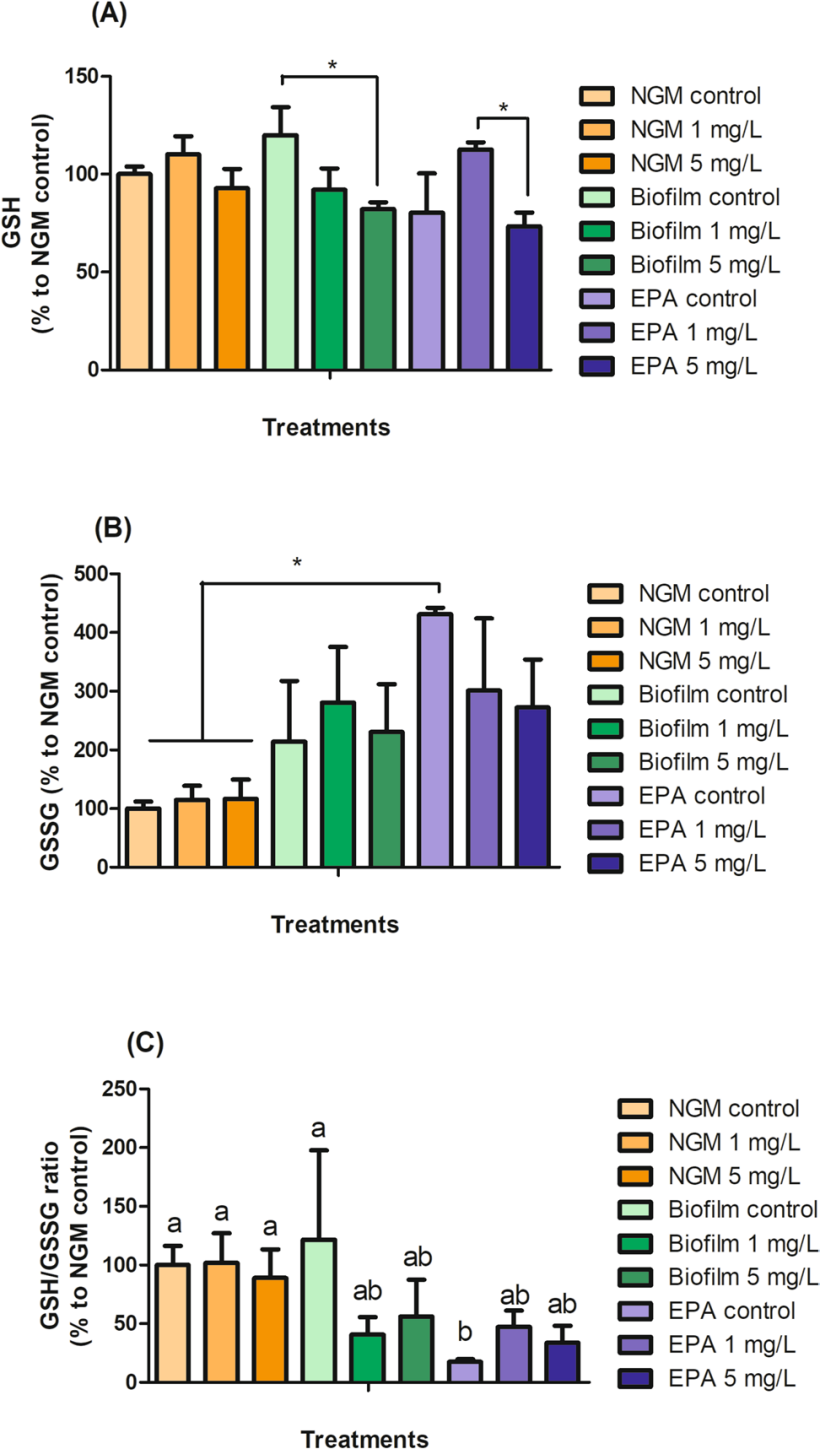
GSH (**A**) and GSSG (**B**) levels and the GSH/GSSG ratio (**C**) of worms exposed to 1 and 5 mg/L AgNP in NGM, Biofilm, and EPA water. The results are expressed as a percentage of NGM control. Different lowercase letters indicate significant differences between the treatments by ANOVA (p < 0.05). Asterisks (*) indicate statistical differences (p < 0.05) after orthogonal contrast

Glutathione (GSH) is a key antioxidant, primarily maintained in its reduced form, with oxidative stress increasing levels of its oxidized form, glutathione disulfide (GSSG) making the GSH-GSSG ratio an important biomarker of oxidative stress (Ferguson and Bridge 2019; Giustarini et al. 2017). Furthermore, GSH has been observed to decrease under AgNP exposure in *C. elegans* (Niu et al. 2023). Our study found no significant changes in GSH levels in worms exposed to cit-AgNP on NGM at any concentration. In the Biofilm, GSH levels decreased at 5 mg/L AgNP, which was consistent with AgNP-induced oxidative stress. In EPA water, GSH increased at 1 mg/L and decreased at 5 mg/L, suggesting an initial antioxidant response (Fig. 4A). GSSG levels were higher in EPA water controls than in NGM controls, with no alterations in the Biofilm group (Fig. 4B). The GSH–GSSG ratio remained unaffected by cit-AgNP exposure, although the EPA water controls showed a lower ratio compared to the NGM and Biofilm control groups, reflecting greater stress impact of the liquid media (EPA water) in worms, in line with DAF-16 data (Fig. 4C). Finally, growth and reproduction, though not directly tied to GSH or GSSG levels, can be sensitive to oxidative stress, which can disrupt GSH/GSSG redox balance, impair somatic cell growth, and affect germline development or gametogenesis, affecting the reproductive capacity in worms (Park et al. 2009; Lee et al. 2023).

#### Ag content in worms

**Fig. 5:**
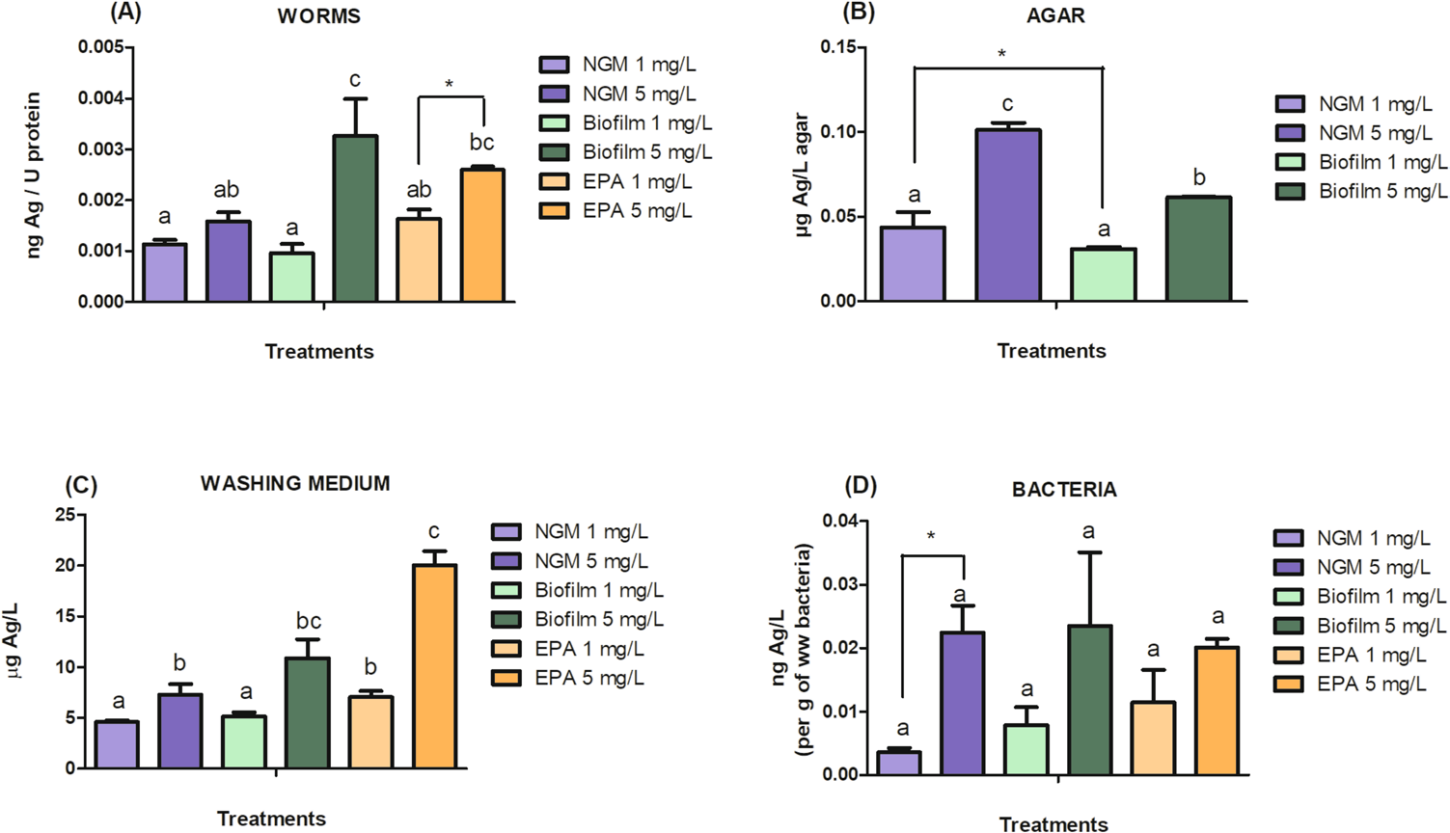
Ag content in worms (**A**) and, Ag distribution in agar (**B**), washing media (**C**), and attached to the remaining bacteria and/or precipitated agglomerates/aggregates (**D**) after worms were exposed to 1 mg/L and 5 mg/L AgNP in NGM, Biofilm, and EPA water. Control groups are not shown because they were below the Ag limit of quantification. Different lowercase letters indicate significant differences between the treatments by ANOVA (p < 0.05). Asterisk (*) indicates statistical differences (p < 0.05) after orthogonal contrast. U protein stands for protein units since the data comes NANODROP equipment

To investigate the influence of the media on Ag bioavailability and its correlation with the toxicity results, we quantified Ag in worms after 52 h. At 1 mg/L, no differences in Ag accumulation in worms were observed among the three media (Fig. 5A). However, at 5 mg/L, worms in the Biofilm group had higher Ag levels than those in the NGM group. Within each medium, the largest increase in Ag content from 1 mg/L to 5 mg/L occurred in Biofilm, followed by EPA water, with no change in NGM. This low Ag content in NGM worms explains the lack of observed toxic effects, while a higher Ag content in EPA water at 5 mg/L correlates with increased toxicity. In Biofilm, despite the high Ag content at 5 mg/L, toxicity did not increase as it did in EPA water, indicating that the amount of Ag content in worms was not solely responsible for the cit-AgNP toxicity. We further explored Ag distribution and found that at 5 mg/L, more Ag migrated into the agar in the NGM, reducing its bioavailability to worms (Fig. 5B). This migration may be due to the precipitation or complexation of Ag with NGM components such as phosphate and chloride (Xiu et al. 2011). This is concerning, as only 15% of studies measure Ag content (Table S2), underscoring how exposure media can significantly mask toxicity outcomes, particularly in NGM. The Ag content in the washing medium was higher in EPA water at 5 mg/L, clearly due to the absence of agar (Fig. 5C). No significant differences in Ag attached to bacteria and/or precipitated agglomerates/aggregates were observed, except in the NGM (Fig. 5D). Finally, as Ag can interact with plastic (such as from Petri dishes used for exposure), a small fraction of AgNP may not have been available to the worms, but this fraction was not quantified (Li et al. 2022). These findings suggest that adding EPA water to NGM (Biofilm) improves Ag bioavailability by preventing its migration into agar compared to regular NGM media alone and improves AgNP stability as observed in the DLS results.

In this study, we observed that medium composition strongly influences AgNP toxicity in *C. elegans*. This effect is linked to the chemical properties of each medium. All liquid media impaired worm fitness, even in the absence of AgNP. When tested without worms, these media (except EPA water) also promoted AgNP agglomeration/aggregation due to their high ionic strength. When all variables were considered, worms exposed in EPA water experienced the most pronounced AgNP-induced toxicity. This effect appears to be largely driven by the inherent stress imposed by the medium itself, rather than the direct toxicological action of AgNP. In contrast, non-liquid media such as NGM and Biofilm were non-detrimental to worm fitness in the absence of AgNP. The presence of agar and nutrients in these media provides great conditions for worm development and fitness, which should not be a surprise since NGM is already used as standard media for *C. elegans* maintenance. Interestingly, in NGM the composition further reduced AgNP toxicity, likely because nanoparticle instability and migration into the agar decreased Ag bioavailability, as reflected by the absence of significant effects on the analyzed parameters. This effect was less pronounced in the Biofilm medium, where Ag remained more bioavailable and worms showed greater sensitivity to their toxicity. This could be related to the Biofilm’s composition, which includes EPA water and maintains higher AgNP stability than NGM, while the thin layer of water in the biofilm helps prevent particle migration into the agar.

It is important to note that comparing different media is inherently challenging, as the liquid fraction in EPA water is much greater than in NGM and Biofilm. To address this, we tried to standardize the amount of AgNP available to worms by using the total volume of bacteria. However, this approach has its own limitations, since AgNP are more diluted in liquid media. This highlights the inherent difficulties in comparing different exposure media and underscores that comparisons between liquid and solid systems should be interpreted with caution due to their susceptibility to error. Additionally, although silver ion release is a key factor in AgNP toxicity, we did not measure Ag dissolution but instead focused on comparing different exposure setups (liquid media, NGM, and Biofilm). Previous studies have shown that dissolution strongly depends on medium conditions (Meyer et al. 2010; Yang et al. 2012, 2019), and our findings provide complementary insights by examining bioaccumulation and effects across these exposure environments. Finally, we should acknowledge that the exposure concentrations were not analytically validated, which represents a limitation of this study. Although we obtained the cit-AgNP directly from the supplier, our interpretation of the toxicological responses relies on the nominal concentrations.

Our results demonstrate that different media elicit distinct AgNP toxicity responses in worms, due to the influence of media on AgNP stability causing the migration of Ag to agar and because of their effects on worm fitness, which can increase their toxicity. Therefore, we encourage researchers to carefully consider the choice of test media and acknowledge their potential impacts on the experimental outcomes. Our findings indicate that Biofilm medium would be an interesting alternative as a suitable medium to maintain worm fitness and be sensitive to AgNP toxicity; however, since there is no standardized protocol or medium to date, reporting bioavailability data should be mandatory to facilitate more accurate comparisons between studies, especially since only 15% of the studies have measured or quantified Ag accumulation. Several factors can affect or cause artifacts in toxicity assays with nanoparticles such as the types of nanoparticles (surface, charge, composition, and size), exposure media (composition and ionic strength), bacteria (concentration and active/inactive), plate shaking, exposure time, and larval stage (Hanna et al. 2018). Therefore, it is imperative to restrain as many variables as possible to ensure the reproducibility of the assays.

## Conclusions

In summary, we demonstrated that all liquid media tested—M9 buffer, K-medium, S-basal, and EPA water—can significantly impact *C. elegans* fitness even in the absence of AgNP. This finding has important implications for the toxicological effects of AgNP on worms, particularly regarding growth, reproduction, and oxidative stress, even in media with low ionic strength such as EPA water. Although the use of NGM medium ensures worms remain in healthy conditions, it leads to an underestimation of AgNP toxicity due to reduced accumulation caused by migration of Ag into the agar and the reduced exposure concentrations compared to liquid media. In contrast, the relatively new Biofilm medium, a combination of NGM and EPA water, appears to offer a practical and balanced alternative, maintaining worm fitness while allowing for a more accurate toxicological response. Overall, this study shows that the choice of test medium significantly influences toxicological outcomes and that incorporating bioavailability or accumulation data is essential for enabling more accurate and reliable comparisons in future studies. Finally, these findings underscore the urgent need for a standardized test medium for toxicological assays involving AgNP in *C. elegans*.

## Supplementary Information

Below is the link to the electronic supplementary material.

ESM 1 (DOCX 2.89 MB)

## Data Availability

Data will be available on reasonable request
