# Supplementary material for: Comparative analysis of nanosilver toxicity in C. elegans: influence of exposure media on accumulation, physiological and biochemical effects
Source: Environ Sci Pollut Res Int. 2026 Jan 22;33(4):1365–76. doi: 10.1007/s11356-025-37339-7 (PMC12901214; doi:10.1007/s11356-025-37339-7)
Supplement: Supplementary file 1 — (DOCX 2.89 MB) [file 11356_2025_37339_MOESM1_ESM.docx]

**SUPPLEMENTARY MATERIAL**

**Table S1.** Molar concentrations of the ions and ionic strength in S-basal, EPA water, M9 buffer, and K-medium (Adapted from Tyne et al., 2013).

| **Element** | **M9 buffer (mM)** | **K-medium (mM)** | **EPA (mM)** | **S-basal (mM)** |
| --- | --- | --- | --- | --- |
| Na | 170 | 222.44 | 1.143 | 100.1 |
| Mg | 0.1 | – | 0.5 |  |
| Al | – | – | - |  |
| K | 22 | 31.656 | 0.054 | 49.83 |
| Ca | – | – | 0.349 |  |
| Fe (III) | – | – | - |  |
| Cl | 85.6 | 254 | 0.054 | 100.1 |
| HCO_3_ | – | – | 1.143 |  |
| SO_4_ | 0.1 | – | 0.849 |  |
| PO_4_ | 64.3 | – | - | 49.09 |
| **Ionic strength** | **234** | **254** | **4.58** | **149.93** |

**Table S2.** Studies that evaluate the toxicity of AgNP in *C. elegans*

| Article Title | Author/Year |
| --- | --- |
| Ecotoxicity of Silver Nanoparticles on the Soil Nematode Caenorhabditis elegans Using Functional Ecotoxicogenomics | (Roh et al., 2009) |
| Intracellular uptake and associated toxicity of silver nanoparticles in Caenorhabditis elegans | (Meyer et al., 2010) |
| Mechanism of Silver Nanoparticle Toxicity Is Dependent on Dissolved Silver and Surface Coating in Caenorhabditis elegans | (Yang et al., 2012) |
| Nano-silver induces dose-response effects on the nematode Caenorhabditis elegans | (Ellegaard-Jensen et al., 2012) |
| Interaction of Silver Nanoparticles with Biological Surfaces of Caenorhabditis elegans | (Kim et al., 2012) |
| Oxidative stress-related PMK-1 P38 MAPK activation as a mechanism for toxicity of silver nanoparticles to reproduction in the nematode Caenorhabditis elegans | (Lim et al., 2012) |
| Involvement of caenohabditis elegans mapk signaling pathways in oxidative stress response induced by silver nanoparticles exposure | (Roh et al., 2012) |
| Nanosilver suppresses growth and induces oxidative damage to DNA in Caenorhabditis elegans | (Hunt et al., 2013) |
| Hypoxia inducible factor-1 (HIF-1)-flavin containing monooxygenase-2 (FMO-2) signaling acts in silver nanoparticles and silver ion toxicity in the nematode, Caenorhabditis elegans | (Eom et al., 2013) |
| Sulfidation of Silver Nanoparticles: Natural Antidote to Their Toxicity | (Levard et al., 2013) |
| Microworms swallow the nanobait: the use of nanocoated microbial cells for the direct delivery of nanoparticles into Caenorhabditis elegans | (Däwlätşina et al., 2013) |
| A new medium for Caenorhabditis elegans toxicology and nanotoxicology studies designed to better reflect natural soil solution conditions | (Tyne et al., 2013) |
| Silver Nanoparticle Behavior, Uptake, and Toxicity in Caenorhabditis elegans: Effects of Natural Organic Matter | (Yang et al., 2014) |
| Effects of Silver Nanoparticles on Oxidative DNA Damage-repair as a Function of p38 MAPK Status: A Comparative Approach Using Human Jurkat T Cells and the Nematode Caenorhabditis elegans | (Chatterjee et al., 2014) |
| Comparative toxicity of silver nanoparticles on oxidative stress and DNA damage in the nematode, Caenorhabditis elegans | (Ahn et al., 2014) |
| Impact of Ag and Al2O3 nanoparticles on soil organisms: In vitro and soil experiments | (Fajardo et al., 2014) |
| SIZE-DEPENDENT IMPACTS OF SILVER NANOPARTICLES ON THE LIFESPAN, FERTILITY, GROWTH, AND LOCOMOTION OF CAENORHABDITIS ELEGANS | (Contreras et al., 2014) |
| Bioactivity of nanosilver in Caenorhabditis elegans: Effects of size, coat, and shape | (Hunt et al., 2014) |
| Enhanced toxicity of silver nanoparticles in transgenic Caenorhabditis elegans expressing amyloidogenic proteins | (Soria et al., 2015) |
| Reducing Environmental Toxicity of Silver Nanoparticles through Shape Control | (Gorka et al., 2015) |
| Impact of sulfidation on the bioavailability and toxicity of silver nanoparticles to Caenorhabditis elegans | (Starnes et al., 2015) |
| Silver nanoparticle toxicity to atlantic killifish (fundulus heteroclitus) and caenorhabditis elegans: a comparison of mesocosm, microcosm, and conventional laboratory studies | (Bone et al., 2015) |
| Hormesis depends upon the life-stage and duration of exposure: Examples for a pesticide and a nanomaterial | (Tyne et al., 2015) |
| Multi-endpoint, high-throughput study of nanomaterial toxicity in Caenorhabditis elegans | (Jung et al., 2015) |
| Effect of natural organic matter on dissolution and toxicity of sulfidized silver nanoparticles to Caenorhabditis elegans | (Collin et al., 2016) |
| Multigenerational exposure to silver ions and silver nanoparticles reveals heightened sensitivity and epigenetic memory in Caenorhabditis elegans | (Schultz et al., 2016) |
| Intracellular trafficking pathways in silver nanoparticle uptake and toxicity in Caenorhabditis elegans | (Maurer et al., 2016) |
| Distinct transcriptomic responses of Caenorhabditis elegans to pristine and sulfidized silver nanoparticles | (Starnes et al., 2016) |
| Insights into the Ecotoxicity of Silver Nanoparticles Transferred from Escherichia coli to Caenorhabditis elegans | (Luo et al., 2016) |
| Nematode-based biomarkers as critical risk indicators on assessing the impact of silver nanoparticles on soil ecosystems | (Yang et al., 2017) |
| Taking the Silver Bullet Colloidal Silver Particles for the Topical Treatment of Biofilm-Related Infections | (Richter et al., 2017) |
| A novel method for assessing the toxicity of silver nanoparticles in Caenorhabditis elegans | (Luo et al., 2017) |
| C-elegans-on-a-chip for in situ and in vivo Ag nanoparticles' uptake and toxicity assay | (Kim et al., 2017) |
| Characterizing the behavior, uptake, and toxicity of NM300K silver nanoparticles in Caenorhabditis elegans | (Kleiven et al., 2018) |
| Developing adverse outcome pathways on silver nanoparticle-induced reproductive toxicity via oxidative stress in the nematode Caenorhabditis elegans using a Bayesian network model | (Jeong et al., 2018) |
| Immune and xenobiotic response crosstalk to chemical exposure by PA01 infection in the nematode Caenorhabditis elegans | (Kim et al., 2018) |
| Biogenic synthesis of silver nanoparticles using Piper betle aqueous extract and evaluation of its anti-quorum sensing and antibiofilm potential against uropathogens with cytotoxic effects: an in vitro and in vivo approach | (Srinivasan et al., 2018) |
| Green synthesized silver nanoparticles demonstrating enhanced in vitro and in vivo antibiofilm activity against Candida spp. | (Muthamil et al., 2018) |
| Effect of ionic strength on bioaccumulation and toxicity of silver nanoparticles in Caenorhabditis elegans | (Yang et al., 2018) |
| Life span-resolved nanotoxicology enables identification of age-associated neuromuscular vulnerabilities in the nematode Caenorhabditis elegans | (Piechulek and von Mikecz, 2018) |
| Influence of soil porewater properties on the fate and toxicity of silver nanoparticles to Caenorhabditis elegans | (Schultz et al., 2018) |
| Genomic mutations after multigenerational exposure of Caenorhabditis elegans to pristine and sulfidized silver nanoparticles | (Wamucho et al., 2019) |
| Multigenerational effects of ecotoxicological interaction between arsenic and silver nanoparticles | (Josende et al., 2019) |
| Effects of ionic strength on physicochemical properties and toxicity of silver nanoparticles | (Yang et al., 2019) |
| The effects of silver nanomaterial shape and size on toxicity to Caenorhabditis elegans in soil media | (Moon et al., 2019) |
| Adaptive tolerance to multigenerational silver nanoparticle (NM300K) exposure by the nematode Caenorhabditis elegans is associated with increased sensitivity to AgNO3 | (Rossbach et al., 2019) |
| UV-induced over time transformation of AgNPs in commercial wound dressings and adverse biological effects on Caenorhabditis elegans | (Nie et al., 2020) |
| Toxicity evaluation of nanocrystalline silver-impregnated coated dressing on the life cycle of worm Caenorhabditis elegans | (Ayech et al., 2020) |
| Antibacterial properties and in vivo studies of tannic acid-stabilized silver-halloysite nanomaterials | (Stavitskaya et al., 2020) |
| Comprehensive phenotyping and transcriptome profiling to study nanotoxicity in C. elegans | (Viau et al., 2020) |
| Chemical transformation and surface functionalisation affect the potential to group nanoparticles for risk assessment | (Schultz et al., 2020) |
| Epigenetic effects induced by silver nanoparticles in Caenorhabditis elegans after multigenerational exposure | (Wamucho et al., 2020) |
| In vivo assessment of silver nanoparticle induced reactive oxygen species reveals tissue specific effects on cellular redox status in the nematode Caenorhabditis elegans | (Rossbach et al., 2020) |
| Protein corona on biogenic silver nanoparticles provides higher stability and protects cells from toxicity in comparison to chemical nanoparticles | (Spagnoletti et al., 2021) |
| Impact of multigenerational exposure to AgNO3 or NM300K Ag NPs on antioxidant defense and oxidative stress in Caenorhabditis elegans | (Rossbach et al., 2021) |
| Toxicity assessment of silver nanoparticles synthesized using endophytic fungi against nosacomial infection | (S. et al., 2021) |
| Neurobehavior and neuron damage following prolonged exposure of silver nanoparticles with/without polyvinylpyrrolidone coating in Caenorhabditis elegans | (Zhang et al., 2021) |
| In vitro and in vivo assessments of inspired Ag/80S bioactive nanocomposites against carbapenem-resistant Klebsiella pneumoniae | (Yang et al., 2021) |
| Estimates of AgNP toxicity thresholds in support of environmental safety policies | (Castro et al., 2022) |
| Colloidal silver combating pathogenic Pseudomonas aeruginosa and MRSA in chronic rhinosinusitis | (Feizi et al., 2021) |
| Antibiofilm efficacy of novel biogenic silver nanoparticles from Terminalia catappa against food-borne Listeria monocytogenes ATCC 15,313 and mechanisms investigation in-vivo and in-vitro | (Muthulakshmi et al., 2022) |
| Silver nanoparticles grafted onto tannic acid-modified halloysite clay eliminated multidrug-resistant Salmonella Typhimurium in a Caenorhabditis elegans model of intestinal infection | (Majumder et al., 2022) |
| Effects of environmental factor fulvic acid on AgNPs food chain delivery and bioavailability | (Luo et al., 2022a) |
| Role of insulin signaling pathway in apoptosis induced by food chain delivery of nano-silver under the action of environmental factors | (Luo et al., 2022b) |
| In Vivo Effects of Silver Nanoparticles on Development, Behavior, and Mitochondrial Function are Altered by Genetic Defects in Mitochondrial Dynamics | (Mello et al., 2022) |
| Silver nanoparticles enhance the efficacy of aminoglycosides against antibiotic-resistant bacteria | (Dove et al., 2022) |
| Global DNA Adenine Methylation in Caenorhabditis elegans after Multigenerational Exposure to Silver Nanoparticles and Silver Nitrate | (Wamucho et al., 2023) |
| Potent anthelmintic activity of a colloidal nano-silver formulation (Silversol®) against the model worm Caenorhabditis elegans | (Gajera et al., 2023) |
| Iron oxide/silver hybrid nanoparticles impair the cholinergic system and cause reprotoxicity in Caenorhabditis elegans | (Silva et al., 2023) |
| Gelatin composite gel particles comprised of in-situ formed zinc oxide and silver nanoparticles with enhanced antibacterial activities via enzymatic degradation | (Hui-Zhong et al., 2023) |
| Unveiling potentially convergent key events related to adverse outcome pathways induced by silver nanoparticles via cross-species omics-scale analysis | (Anh et al., 2023) |
| Comparative oxidative damages induced by silver nanoparticles with different sizes and coatings in Caenorhabditis elegans | (Niu et al., 2023) |
| Synthesis, Characterization, and Antimicrobial and Nematicidal Activities of Chitosan-Based Silver-Doped Titanium Dioxide | (Usman Khan et al., 2023) |
| Effects of Multiple Stressors, Pristine or Sulfidized Silver Nanomaterials, and a Pathogen on a Model Soil Nematode Caenorhabditis elegans | (Cochran et al., 2024) |
| Comparison of silver nanoparticles and agno3 toxic effects on Caenorhabditis elegans with a genetic disorder of copper metabolism | (Samuseva et al., 2024) |
| Biogenic silver nanoparticles from Simarouba glauca DC leaf extract: Synthesis, characterization, and anticancer efficacy in lung cancer cells with protective effects in Caenorhabditis elegans | (Bhavi et al., 2024) |
| Green synthesized silver nanoparticles from Phoenix dactylifera synergistically interact with bioactive extract of Punica granatum against bacterial virulence and biofilm development | (Samreen et al., 2024) |
| In Situ Preparation of Dehydrodieugenol-Loaded Silver Nanoparticles and their Antischistosomal Activity | (Oliveira et al., 2024) |
| Fabrication and characterisation of human gut microbiome derived exopolysaccharide mediated silver nanoparticles – An in-vitro and in-vivo approach of Bio-Pm-AgNPs targeting Vibrio cholerae | (Ravindran et al., 2024) |
| Açaí (Euterpe oleracea Mart.) green synthesis of silver nanoparticles: antimicrobial efficacy and ecotoxicological assessment | (Taipe Huisa et al., 2024) |
| The effect of Clitoria ternatea L. flowers-derived silver nanoparticles on A549 and L-132 human cell lines and their antibacterial efficacy in Caenorhabditis elegans in vivo | (Singh et al., 2025) |
| Metal copper and silver revealed potent antimicrobial activity for treating Caenorhabditis elegans infected with carbapenemase-producing Klebsiella pneumonia | (Cao et al., 2024) |

***Methodology:** Studies obtained using the following keywords: *((Caenorhabditis elegans) or (C. elegans)) AND ((silver nanoparticles) OR NANOSILVER)*. Databases used were Science Direct and Web of Science, with last access on 31/12/2024. The total number of studies included is 82 (after excluding duplicates, and out of scope)**.**

**Table S3.** LC-MS/MS parameters

|  | Parameter | Working conditions |
| --- | --- | --- |
| LC (Agilent 1260 Infinity II) | Injection volume: | 5 µL |
|  | Mobile phase A: | dH_2_O + 0.1% FA |
|  | Mobile phase B: | ACN + 0.1% FA |
|  | Column temperature: | 20° C |
|  | Column: | Waters Atlantis T3 |
| MS/MS (Sciex QTrap 6500+) 🡪 low mass | Acquisition Method Time | 5 min |
|  | Scan type | MRM |
|  | CUR | 40 |
|  | CAD | Medium |
|  | IS | 4000 |
|  | TEM | 550 °C |
|  | GS1 | 40 |
|  | GS2 | 40 |
|  | EP | 10 |
|  | Dwell time | 35 ms |

**Table S4.** ICP-OES parameters

| Parameter | Working conditions |
| --- | --- |
| Plasma power [W] | 1500 |
| Flow cooling gas [L/min] | 8 |
| Flow auxiliary gas [L/min] | 0.2 |
| Flow nebulizer gas [L/min] | 0.7 |
| Nebulizer | MicroMist® |
| Torch alignment | Axial |
| Rinse time [s] | 40 |
| Stabilization time [s] | 30 |
| Sample amount [mL] | 3 mL |
| Software analysis | Syngistix |
| Element wavelengths [nm] | Mn: 267.61  Cu: 327.33  Ag: 328.068/338.29 |

**Fig. S1** Types of exposure media used in nanotoxicity studies involving silver nanoparticles in *C. elegans* (n=82). See Table S2

**Fig. S2** Exposure media used over the years from the first publication registered in 2009 until 2024 (n = 82). See Table S2

**Fig. S3** Transmission electron micrograph of citrate-silver nanoparticles


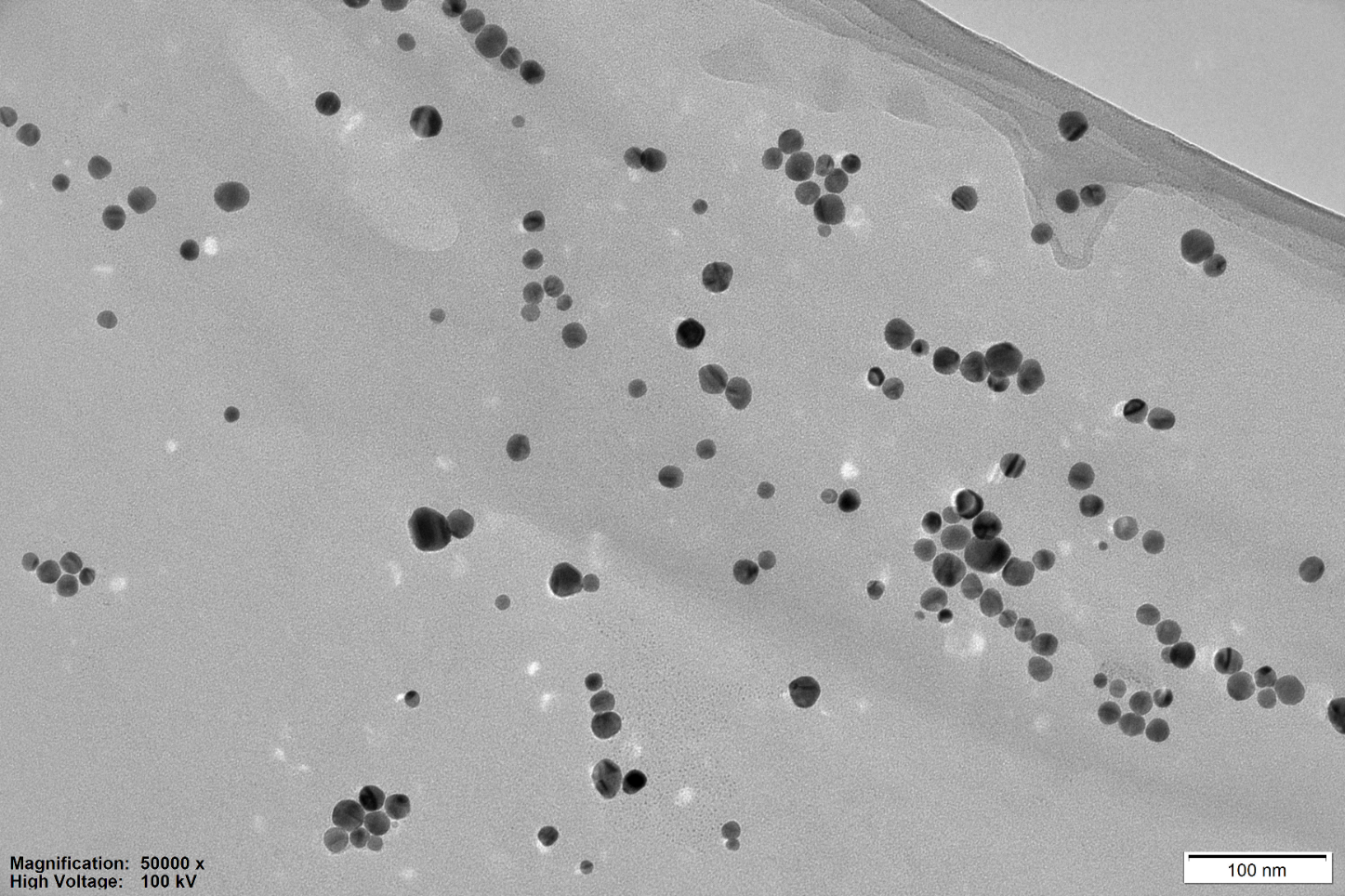


**Fig. S4** Vulvar abnormalities observed in worms treated with 5 mg/L AgNP in NGM (A, B) and Biofilm (C, D)


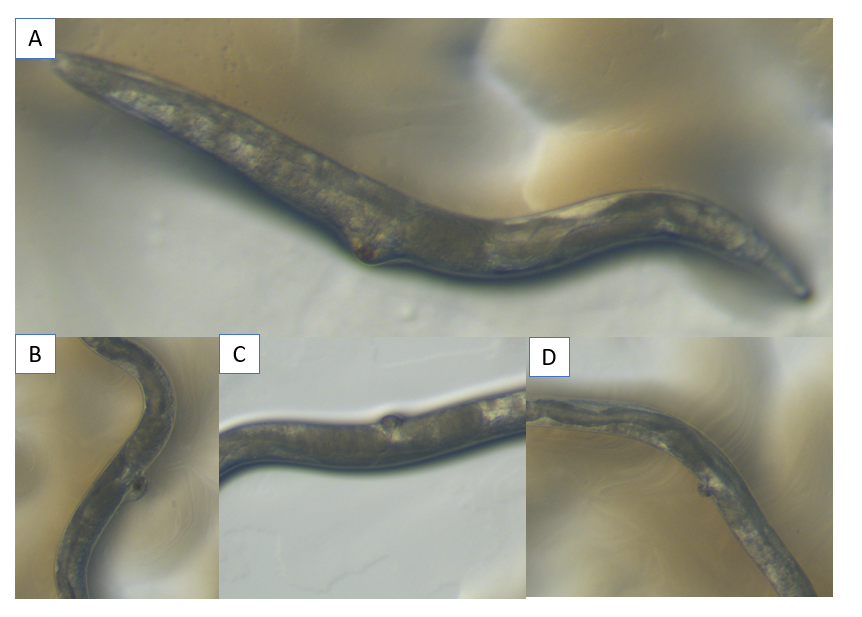


**REFERENCES**

Ahn, J.-M., Eom, H.-J., Yang, X., Meyer, J.N., Choi, J. (2014). Comparative toxicity of silver nanoparticles on oxidative stress and DNA damage in the nematode, *Caenorhabditis elegans*. Chemosphere 108, 343–352. https://doi.org/10.1016/j.chemosphere.2014.01.078

Anh, N.H., Min, Y.J., Thi My Nhung, T., Long, N.P., Han, S., Kim, S.J., Jung, C.W., Yoon, Y.C., Kang, Y.P., Park, S.K., Kwon, S.W., (2023). Unveiling potentially convergent key events related to adverse outcome pathways induced by silver nanoparticles via cross-species omics-scale analysis. J. Hazard. Mater. 459, 132208. https://doi.org/10.1016/j.jhazmat.2023.132208

Ayech, A., Josende, M.E., Ventura-Lima, J., Ruas, C., Gelesky, M.A., Ale, A., Cazenave, J., Galdopórpora, J.M., Desimone, M.F., Duarte, M., Halicki, P., Ramos, D., Carvalho, L.M., Leal, G.C., Monserrat, J.M. (2020). Toxicity evaluation of nanocrystalline silver-impregnated coated dressing on the life cycle of worm Caenorhabditis elegans. Ecotoxicol. Environ. Saf. 197, 110570. https://doi.org/10.1016/j.ecoenv.2020.110570

Bhavi, S.M., Padti, A.C., Thokchom, B., Singh, S.R., Bhat, S.S., Bajire, S.K., Shastry, R.P., Srinath, B.S., Gummani, S.S., Harini, B.P., Yarajarla, R.B. (2024). Biogenic silver nanoparticles from *Simarouba glauca* DC leaf extract: Synthesis, characterization, and anticancer efficacy in lung cancer cells with protective effects in *Caenorhabditis elegans*. Nano TransMed 3, 100052. https://doi.org/10.1016/j.ntm.2024.100052

Bone, A.J., Matson, C.W., Colman, B.P., Yang, X., Meyer, J.N., Di Giulio, R.T. (2015). Silver nanoparticle toxicity to Atlantic killifish (*Fundulus heteroclitus*) and *Caenorhabditis elegans*: a comparison of mesocosm, microcosm, and conventional laboratory studies. Environ. Toxicol. Chem. 34, 275–282. https://doi.org/10.1002/etc.2806

Cao, L., Tan, L., Li, L. (2024). Metal copper and silver revealed potent antimicrobial activity for treating *Caenorhabditis elegans* infected with carbapenemase-producing *Klebsiella pneumonia*. Am. J. Transl. Res. 16, 2011–2023. https://doi.org/10.62347/DIEO8870

Castro, V.L., Jonsson, C.M., Silva, M.S.G.M., Castanha, R., Vallim, J.H., da Silva, L.A.G., de Oliveira, R.M.D., Correa, D.S., Ferreira, M.D. (2022). Estimates of AgNP toxicity thresholds in support of environmental safety policies. J. Nanoparticle Res. 24, 9. https://doi.org/10.1007/s11051-021-05357-4

Chatterjee, N., Eom, H.J., Choi, J. (2014). Effects of silver nanoparticles on oxidative DNA damage–repair as a function of p38 MAPK status: A comparative approach using human Jurkat T cells and the nematode *Caenorhabditis elegans*. Environ. Mol. Mutagen. 55, 122–133. https://doi.org/10.1002/em.21844

Cochran, J.P., Ngy, P., Unrine, J.M., Matocha, C.J., Tsyusko, O.V. (2024). Effects of multiple stressors, pristine or sulfidized silver nanomaterials, and a pathogen on a model soil nematode *Caenorhabditis elegans.* Nanomaterials 14, 913. https://doi.org/10.3390/nano14110913

Collin, B., Tsyusko, O.V., Starnes, D.L., Unrine, J.M. (2016). Effect of natural organic matter on dissolution and toxicity of sulfidized silver nanoparticles to *Caenorhabditis elegans*. Environ. Sci. Nano 3, 728–736. https://doi.org/10.1039/C6EN00095A

Contreras, E.Q., Puppala, H.L., Escalera, G., Zhong, W., Colvin, V.L. (2014). Size-dependent impacts of silver nanoparticles on the lifespan, fertility, growth, and locomotion of *Caenorhabditis elegans*. Environ. Toxicol. Chem. 33, 2716–2723. https://doi.org/10.1002/etc.2705

Däwlätşina, G.I., Minullina, R.T., Fakhrullin, R.F. (2013). Microworms swallow the nanobait: the use of nanocoated microbial cells for the direct delivery of nanoparticles into *Caenorhabditis elegans.* Nanoscale 5, 11761–11769. https://doi.org/10.1039/c3nr03905f

Dove, A.S., Dzurny, D.I., Dees, W.R., Qin, N., Nunez Rodriguez, C.C., Alt, L.A., Ellward, G.L., Best, J.A., Rudawski, N.G., Fujii, K., Czyż, D.M. (2022). Silver nanoparticles enhance the efficacy of aminoglycosides against antibiotic-resistant bacteria. Front. Microbiol. 13, 1064095. https://doi.org/10.3389/fmicb.2022.1064095

Ellegaard-Jensen, L., Jensen, K.A., Johansen, A. (2012). Nano-silver induces dose-response effects on the nematode *Caenorhabditis elegans*. Ecotoxicol. Environ. Saf. 80, 216–223. https://doi.org/10.1016/j.ecoenv.2012.03.003

Eom, H.-J., Ahn, J.-M., Kim, Y., Choi, J. (2013). Hypoxia inducible factor-1 (HIF-1)–flavin containing monooxygenase-2 (FMO-2) signaling acts in silver nanoparticles and silver ion toxicity in the nematode, *Caenorhabditis elegans*. Toxicol. Appl. Pharmacol. 270, 106–113. https://doi.org/10.1016/j.taap.2013.03.028

Fajardo, C., Saccà, M.L., Costa, G., Nande, M., Martin, M. (2014). Impact of Ag and Al2O3 nanoparticles on soil organisms: In vitro and soil experiments. Sci. Total Environ. 473–474, 254–261. https://doi.org/10.1016/j.scitotenv.2013.12.043

Feizi, S., Cooksley, C.M., Bouras, G.S., Prestidge, C.A., Coenye, T., Psaltis, A.J., Wormald, P.-J., Vreugde, S. (2021). Colloidal silver combating pathogenic *Pseudomonas aeruginosa* and MRSA in chronic rhinosinusitis. Colloids Surf. B Biointerfaces 202, 111675. https://doi.org/10.1016/j.colsurfb.2021.111675

Gajera, G., Godse, C., DeSouza, A., Mehta, D., Kothari, V., 2023. Potent anthelmintic activity of a colloidal nano-silver formulation (Silversol®) against the model worm Caenorhabditis elegans. BMC Res. Notes 16, 130. https://doi.org/10.1186/s13104-023-06392-1

Gorka, D.E., Osterberg, J.S., Gwin, C.A., Colman, B.P., Meyer, J.N., Bernhardt, E.S., Gunsch, C.K., DiGulio, R.T., Liu, J. (2015). Reducing environmental toxicity of silver nanoparticles through shape control. Environ. Sci. Technol. 49, 10093–10098. https://doi.org/10.1021/acs.est.5b01711

Hui-Zhong, Z., Chen, Y.-F., Ya-Chu, Y., Cheng-Rung, H., Yi-Sheng, J., Chang-Shi, C., Jan, J.-S. (2023). Gelatin composite gel particles comprised of in-situ formed zinc oxide and silver nanoparticles with enhanced antibacterial activities via enzymatic degradation. Colloids Surf. Physicochem. Eng. Asp. 678, 132509. https://doi.org/10.1016/j.colsurfa.2023.132509

Hunt, P.R., Keltner, Z., Gao, X., Oldenburg, S.J., Bushana, P., Olejnik, N., Sprando, R.L. (2014). Bioactivity of nanosilver in *Caenorhabditis elegans*: Effects of size, coat, and shape. Toxicol. Rep. 1, 923–944. https://doi.org/10.1016/j.toxrep.2014.10.020

Hunt, P.R., Marquis, B.J., Tyner, K.M., Conklin, S., Olejnik, N., Nelson, B.C., Sprando, R.L. (2013). Nanosilver suppresses growth and induces oxidative damage to DNA in *Caenorhabditis elegans*. J. Appl. Toxicol. 33, 1131–1142. https://doi.org/10.1002/jat.2872

Jeong, J., Song, T., Chatterjee, N., Choi, I., Cha, Y.K., Choi, J. (2018). Developing adverse outcome pathways on silver nanoparticle-induced reproductive toxicity via oxidative stress in the nematode *Caenorhabditis elegans* using a Bayesian network model. Nanotoxicology 12, 1182–1197. https://doi.org/10.1080/17435390.2018.1529835

Josende, M.E., Nunes, S.M., Müller, L., dos Santos Francisco, W., Gelesky, M.A., Monserrat, J.M., Ventura-Lima, J. (2019). Multigenerational effects of ecotoxicological interaction between arsenic and silver nanoparticles. Sci. Total Environ. 696, 133947. https://doi.org/10.1016/j.scitotenv.2019.133947

Jung, S.-K., Qu, X., Aleman-Meza, B., Wang, T., Riepe, C., Liu, Z., Li, Q., Zhong, W. (2015). Multi-endpoint, high-throughput study of nanomaterial toxicity in *Caenorhabditis elegans*. Environ. Sci. Technol. 49, 2477–2485. https://doi.org/10.1021/es5056462

Kim, J.H., Lee, S.H., Cha, Y.J., Hong, S.J., Chung, S.K., Park, T.H., Choi, S.S. (2017). *C. elegans*-on-a-chip for in situ and in vivo Ag nanoparticles’ uptake and toxicity assay. Sci. Rep. 7, 40225. https://doi.org/10.1038/srep40225

Kim, S.W., Nam, S.-H., An, Y.-J., (2012). Interaction of silver nanoparticles with biological surfaces of *Caenorhabditis elegans.* Ecotoxicol. Environ. Saf. 77, 64–70. https://doi.org/10.1016/j.ecoenv.2011.10.023

Kim, Y., Choudhry, Q.N., Chatterjee, N., Choi, J. (2018). Immune and xenobiotic response crosstalk to chemical exposure by PA01 infection in the nematode *Caenorhabditis elegans.* Chemosphere 210, 1082–1090. https://doi.org/10.1016/j.chemosphere.2018.07.031

Kleiven, M., Rossbach, L.M., Gallego-Urrea, J.A., Brede, D.A., Oughton, D.H., Coutris, C., 2018. Characterizing the behavior, uptake, and toxicity of NM300K silver nanoparticles in *Caenorhabditis elegans*. Environ. Toxicol. Chem. 37, 1799–1810. https://doi.org/10.1002/etc.4144

Levard, C., Hotze, E.M., Colman, B.P., Dale, A.L., Truong, L., Yang, X.Y., Bone, A.J., Brown, G.E., Tanguay, R.L., Di Giulio, R.T., Bernhardt, E.S., Meyer, J.N., Wiesner, M.R., Lowry, G.V. (2013) Sulfidation of silver nanoparticles: natural antidote to their toxicity. Environ. Sci. Technol. 47, 13440–13448. https://doi.org/10.1021/es403527n

Lim, D., Roh, J.-Y., Eom, H.-J., Choi, J.-Y., Hyun, J., Choi, J. (2012). Oxidative stress-related PMK-1 P38 MAPK activation as a mechanism for toxicity of silver nanoparticles to reproduction in the nematode *Caenorhabditis elegans*. Environ. Toxicol. Chem. 31, 585–592. https://doi.org/10.1002/etc.1706

Luo, X., Xu, S., Yang, Y., Li, L., Chen, S., Xu, A., Wu, L. (2016). Insights into the ecotoxicity of silver nanoparticles transferred from *Escherichia coli* to *Caenorhabditis elegans*. Sci. Rep. 6, 36465. https://doi.org/10.1038/srep36465

Luo, X., Xu, S., Yang, Y., Zhang, Y., Wang, S., Chen, S., Xu, A., Wu, L. (2017). A novel method for assessing the toxicity of silver nanoparticles in *Caenorhabditis elegans*. Chemosphere 168, 648–657. https://doi.org/10.1016/j.chemosphere.2016.11.011

Luo, X., Zhang, Y., Fu, X., Liu, N. (2022a). Effects of environmental factor fulvic acid on AgNPs food chain delivery and bioavailability. Comp. Biochem. Physiol. Toxicol. Pharmacol. CBP 258, 109369. https://doi.org/10.1016/j.cbpc.2022.109369

Luo, X., Zhang, Y., Lu, C., Zhang, J. (2022b). Role of insulin signaling pathway in apoptosis induced by food chain delivery of nano-silver under the action of environmental factors. Comp. Biochem. Physiol. Toxicol. Pharmacol. CBP 261, 109429. https://doi.org/10.1016/j.cbpc.2022.109429

Majumder, S., Viau, C., Brar, A., Xia, J., George, S. (2022). Silver nanoparticles grafted onto tannic acid-modified halloysite clay eliminated multidrug-resistant *Salmonella Typhimurium* in a *Caenorhabditis elegans* model of intestinal infection. Appl. Clay Sci. 228, 106569. https://doi.org/10.1016/j.clay.2022.106569

Maurer, L.L., Yang, X., Schindler, A.J., Taggart, R.K., Jiang, C., Hsu-Kim, H., Sherwood, D.R., Meyer, J.N. (2016). Intracellular trafficking pathways in silver nanoparticle uptake and toxicity in *Caenorhabditis elegans*. Nanotoxicology 10, 831–835. https://doi.org/10.3109/17435390.2015.1110759

Mello, D.F., Maurer, L.L., Ryde, I.T., Songr, D.H., Marinakos, S.M., Jiang, C., Wiesner, M.R., Hsu-Kim, H., Meyer, J.N. (2022). In vivo effects of silver nanoparticles on development, behavior, and mitochondrial function are altered by genetic defects in mitochondrial dynamics. Environ. Sci. Technol. 56, 1113–1124. https://doi.org/10.1021/acs.est.1c05915

Meyer, J.N., Lord, C.A., Yang, X.Y., Turner, E.A., Badireddy, A.R., Marinakos, S.M., Chilkoti, A., Wiesner, M.R., Auffan, M. (2010). Intracellular uptake and associated toxicity of silver nanoparticles in *Caenorhabditis elegans*. Aquat. Toxicol., Aquatic Toxicology of Nanomaterials 100, 140–150. https://doi.org/10.1016/j.aquatox.2010.07.016

Moon, J., Kwak, J.I., An, Y.-J. (2019). The effects of silver nanomaterial shape and size on toxicity to *Caenorhabditis elegans* in soil media. Chemosphere 215, 50–56. https://doi.org/10.1016/j.chemosphere.2018.09.177

Muthamil, S., Devi, V.A., Balasubramaniam, B., Balamurugan, K., Pandian, S.K. (2018). Green synthesized silver nanoparticles demonstrating enhanced in vitro and in vivo antibiofilm activity against *Candida spp*. J. Basic Microbiol. 58, 343–357. https://doi.org/10.1002/jobm.201700529

Muthulakshmi, L., Suganya, K., Murugan, M., Annaraj, J., Duraipandiyan, V., Farraj, D., Elshikh, M., Juliet, A., Pasupuleti, M., Arockiaraj, J. (2022). Antibiofilm efficacy of novel biogenic silver nanoparticles from *Terminalia catappa* against food-borne *Listeria monocytogenes ATCC 15313* and mechanisms investigation in-vivo and in-vitro. J. King Saud Univ. - Sci. 34, 102083. https://doi.org/10.1016/j.jksus.2022.102083

Nie, Y., Wang, Jingjing, Dai, H., Wang, Juan, Wang, M., Cheng, L., Yang, Z., Chen, S., Zhao, G., Wu, L., Xu, A. (2020). UV-induced over time transformation of AgNPs in commercial wound dressings and adverse biological effects on *Caenorhabditis elegans*. NanoImpact 17, 100193. https://doi.org/10.1016/j.impact.2019.100193

Niu, S., Wang, J., Chang, X., Shang, M., Guo, M., Sun, Z., Li, Y., Xue, Y. (2023). Comparative oxidative damages induced by silver nanoparticles with different sizes and coatings in *Caenorhabditis elegans.* Toxicol. Res. 12, 833–842. https://doi.org/10.1093/toxres/tfad074

Oliveira, L.V.F., Camilo, F.F., Soares, M.G., Cajas, R.A., Cirino, M.E., de Moraes, J., Lago, J.H.G. (2024). In situ preparation of dehydrodieugenol-loaded silver nanoparticles and their antischistosomal activity. Chem. Biodivers. 21, e202301929. https://doi.org/10.1002/cbdv.202301929

Piechulek, A., von Mikecz, A. (2018). Life span-resolved nanotoxicology enables identification of age-associated neuromuscular vulnerabilities in the nematode *Caenorhabditis elegans*. Environ. Pollut. 233, 1095–1103. https://doi.org/10.1016/j.envpol.2017.10.012

Ravindran, D.R., Kannan, S., Marudhamuthu, M. (2024). Fabrication and characterisation of human gut microbiome derived exopolysaccharide mediated silver nanoparticles - An in-vitro and in-vivo approach of Bio-Pm-AgNPs targeting *Vibrio cholerae*. Int. J. Biol. Macromol. 256, 128406. https://doi.org/10.1016/j.ijbiomac.2023.128406

Richter, K., Facal, P., Thomas, N., Vandecandelaere, I., Ramezanpour, M., Cooksley, C., Prestidge, C.A., Coenye, T., Wormald, P.-J., Vreugde, S. (2017). Taking the silver bullet colloidal silver particles for the topical treatment of biofilm-related infections. ACS Appl. Mater. Interfaces 9, 21631–21638. https://doi.org/10.1021/acsami.7b03672

Roh, J., Sim, S.J., Yi, J., Park, K., Chung, K.H., Ryu, D., Choi, J. (2009). Ecotoxicity of silver nanoparticles on the soil nematode *Caenorhabditis elegans* using functional ecotoxicogenomics. Environ. Sci. Technol. 43, 3933–3940. https://doi.org/10.1021/es803477u

Roh, J.-Y., Eom, H.-J., Choi, J., 2012. Involvement of *Caenorhabditis elegans* MAPK signaling pathways in oxidative stress response induced by silver nanoparticles exposure. Toxicol. Res. 28, 19–24. https://doi.org/10.5487/TR.2012.28.1.019

Rossbach, L.M., Maremonti, E., Eide, D.M., Oughton, D.H., Brede, D.A. (2019). Adaptive tolerance to multigenerational silver nanoparticle (NM300K) exposure by the nematode *Caenorhabditis elegans* is associated with increased sensitivity to AgNO3. Nanotoxicology 13, 527–542. https://doi.org/10.1080/17435390.2018.1557272

Rossbach, L.M., Oughton, D.H., Maremonti, E., Coutris, C., Brede, D.A. (2020). In vivo assessment of silver nanoparticle induced reactive oxygen species reveals tissue specific effects on cellular redox status in the nematode *Caenorhabditis elegans*. Sci. Total Environ. 721, 137665. https://doi.org/10.1016/j.scitotenv.2020.137665

Rossbach, L.M., Oughton, D.H., Maremonti, E., Eide, D.M., Brede, D.A. (2021). Impact of multigenerational exposure to AgNO3 or NM300K Ag NPs on antioxidant defense and oxidative stress in *Caenorhabditis elegans*. Ecotoxicol. Environ. Saf. 216, 112178. https://doi.org/10.1016/j.ecoenv.2021.112178

S., R., M., S.A., D., M., C., R., N., S.K., S., H. (2021). Toxicity assessment of silver nanoparticles synthesized using endophytic fungi against nosacomial infection. Inorg. Nano-Met. Chem. 51, 1080–1085. https://doi.org/10.1080/24701556.2020.1814332

Samreen, Ahmad, I., Khan, S.A., Naseer, A., Nazir, A. (2024). Green synthesized silver nanoparticles from *Phoenix dactylifera* synergistically interact with bioactive extract of *Punica granatum* against bacterial virulence and biofilm development. Microb. Pathog. 192, 106708. https://doi.org/10.1016/j.micpath.2024.106708

Samuseva, P.D., Tschukina, A.D., Baikina, S.A., Mekhova, A.A. (2024). Comparison of silver nanoparticles and AgNO_3_ toxic effects on *Caenorhabditis elegans* with a genetic disorder of copper metabolism. Med. Acad. J. 24, 131–136. https://doi.org/10.17816/MAJ630331

Schultz, C.L., Adams, J., Jurkschat, K., Lofts, S., Spurgeon, D.J. (2020). Chemical transformation and surface functionalisation affect the potential to group nanoparticles for risk assessment. Environ. Sci. Nano 7, 3100–3107. https://doi.org/10.1039/D0EN00578A

Schultz, C.L., Lahive, E., Lawlor, A., Crossley, A., Puntes, V., Unrine, J.M., Svendsen, C., Spurgeon, D.J., 2018. Influence of soil porewater properties on the fate and toxicity of silver nanoparticles to Caenorhabditis elegans. Environ. Toxicol. Chem. 37, 2609–2618. https://doi.org/10.1002/etc.4220

Schultz, C.L., Wamucho, A., Tsyusko, O.V., Unrine, J.M., Crossley, A., Svendsen, C., Spurgeon, D.J. (2016). Multigenerational exposure to silver ions and silver nanoparticles reveals heightened sensitivity and epigenetic memory in *Caenorhabditis elegans*. Proc. Biol. Sci. 283, 20152911. https://doi.org/10.1098/rspb.2015.2911

Silva, A.C., dos Santos, A.G.R., Pieretti, J.C., Rolim, W.R., Seabra, A.B., Ávila, D.S. (2023). Iron oxide/silver hybrid nanoparticles impair the cholinergic system and cause reprotoxicity in *Caenorhabditis elegans*. Food Chem. Toxicol. 179, 113945. https://doi.org/10.1016/j.fct.2023.113945

Singh, S.R., Kittur, B., Bhavi, S.M., Thokchom, B., Padti, A.C., Bhat, S.S., Bajire, S.K., Shastry, R.P., Srinath, B.S., Sillanpää, M., Harini, B.P., Yarajarla, R.B. (2025). The effect of *Clitoria ternatea* L. flowers-derived silver nanoparticles on A549 and L-132 human cell lines and their antibacterial efficacy in *Caenorhabditis elegans* in vivo. Hybrid Adv. 8, 100359. https://doi.org/10.1016/j.hybadv.2024.100359

Soria, C., Coccini, T., De Simone, U., Marchese, L., Zorzoli, I., Giorgetti, S., Raimondi, S., Mangione, P.P., Ramat, S., Bellotti, V., Manzo, L., Stoppini, M. (2015). Enhanced toxicity of silver nanoparticles in transgenic *Caenorhabditis elegans* expressing amyloidogenic proteins. Amyloid Int. J. Exp. Clin. Investig. Off. J. Int. Soc. Amyloidosis 22, 221–228. https://doi.org/10.3109/13506129.2015.1077216

Spagnoletti, F.N., Kronberg, F., Spedalieri, C., Munarriz, E., Giacometti, R. (2021). Protein corona on biogenic silver nanoparticles provides higher stability and protects cells from toxicity in comparison to chemical nanoparticles. J. Environ. Manage. 297, 113434. https://doi.org/10.1016/j.jenvman.2021.113434

Srinivasan, R., Vigneshwari, L., Rajavel, T., Durgadevi, R., Kannappan, A., Balamurugan, K., Pandima Devi, K., Veera Ravi, A. (2018). Biogenic synthesis of silver nanoparticles using *Piper betle* aqueous extract and evaluation of its anti-quorum sensing and antibiofilm potential against uropathogens with cytotoxic effects: an in vitro and in vivo approach. Environ. Sci. Pollut. Res. 25, 10538–10554. https://doi.org/10.1007/s11356-017-1049-0

Starnes, D.L., Lichtenberg, S.S., Unrine, J.M., Starnes, C.P., Oostveen, E.K., Lowry, G.V., Bertsch, P.M., Tsyusko, O.V. (2016). Distinct transcriptomic responses of *Caenorhabditis elegans* to pristine and sulfidized silver nanoparticles. Environ. Pollut. Barking Essex 1987 213, 314–321. https://doi.org/10.1016/j.envpol.2016.01.020

Starnes, D.L., Unrine, J.M., Starnes, C.P., Collin, B.E., Oostveen, E.K., Ma, R., Lowry, G.V., Bertsch, P.M., Tsyusko, O.V. (2015). Impact of sulfidation on the bioavailability and toxicity of silver nanoparticles to *Caenorhabditis elegans*. Environ. Pollut. 196, 239–246. https://doi.org/10.1016/j.envpol.2014.10.009

Stavitskaya, A., Shakhbazova, C., Cherednichenko, Y., Nigamatzyanova, L., Fakhrullina, G., Khaertdinov, N., Kuralbayeva, G., Filimonova, A., Vinokurov, V., Fakhrullin, R. (2020). Antibacterial properties and in vivo studies of tannic acid-stabilized silver–halloysite nanomaterials. Clay Miner. 55, 112–119. https://doi.org/10.1180/clm.2020.17

Taipe Huisa, A.J., Estrella Josende, M., Gelesky, M.A., Fernandes Ramos, D., López, G., Bernardi, F., Monserrat, J.M. (2024). Açaí (*Euterpe oleracea Mart*.) green synthesis of silver nanoparticles: antimicrobial efficacy and ecotoxicological assessment. Environ. Sci. Pollut. Res. 31, 12005–12018. https://doi.org/10.1007/s11356-024-31949-3

Tyne, W., Little, S., Spurgeon, D.J., Svendsen, C. (2015). Hormesis depends upon the life-stage and duration of exposure: Examples for a pesticide and a nanomaterial. Ecotoxicol. Environ. Saf. 120, 117–123. https://doi.org/10.1016/j.ecoenv.2015.05.024

Tyne, W., Lofts, S., Spurgeon, D.J., Jurkschat, K., Svendsen, C. (2013). A new medium for *Caenorhabditis elegans* toxicology and nanotoxicology studies designed to better reflect natural soil solution conditions. Environ. Toxicol. Chem. 32, 1711–1717. https://doi.org/10.1002/etc.2247

Usman Khan, M., Rehman, W., Bibi, S., Alanazi, M.M., Alanazi, A.S., Rasheed, L., Khan, S., Tariq Gillani, S.U., Tauqeer, A. (2023). Synthesis, characterization, and antimicrobial and nematicidal activities of chitosan-based silver-doped titanium dioxide. ACS Omega 8, 19341–19350. https://doi.org/10.1021/acsomega.3c00068

Viau, C., Haçariz, O., Karimian, F., Xia, J. (2020). Comprehensive phenotyping and transcriptome profiling to study nanotoxicity in *C. elegans*. PeerJ 8, e8684. https://doi.org/10.7717/peerj.8684

Wamucho, A., Heffley, A., Tsyusko, O.V. (2020). Epigenetic effects induced by silver nanoparticles in *Caenorhabditis elegans* after multigenerational exposure. Sci. Total Environ. 725, 138523. https://doi.org/10.1016/j.scitotenv.2020.138523

Wamucho, A., Unrine, J., May, J., Tsyusko, O. (2023). Global DNA adenine methylation in *Caenorhabditis elegans* after multigenerational exposure to silver nanoparticles and silver nitrate. Int. J. Mol. Sci. 24, 6168. https://doi.org/10.3390/ijms24076168

Wamucho, A., Unrine, J.M., Kieran, T.J., Glenn, T.C., Schultz, C.L., Farman, M., Svendsen, C., Spurgeon, D.J., Tsyusko, O.V. (2019). Genomic mutations after multigenerational exposure of *Caenorhabditis elegans* to pristine and sulfidized silver nanoparticles. Environ. Pollut. 254, 113078. https://doi.org/10.1016/j.envpol.2019.113078

Yang, T.-Y., Hsieh, Y.-J., Lu, P.-L., Lin, L., Wang, L.-C., Wang, H.-Y., Tsai, T.-H., Shih, C.-J., Tseng, S.-P. (2021). In vitro and in vivo assessments of inspired Ag/80S bioactive nanocomposites against carbapenem-resistant *Klebsiella pneumoniae*. Mater. Sci. Eng. C Mater. Biol. Appl. 125, 112093. https://doi.org/10.1016/j.msec.2021.112093

Yang, X., Gondikas, A.P., Marinakos, S.M., Auffan, M., Liu, J., Hsu-Kim, H., Meyer, J.N. (2012). Mechanism of silver nanoparticle toxicity is dependent on dissolved silver and surface coating in *Caenorhabditis elegans*. Environ. Sci. Technol. 46, 1119–1127. https://doi.org/10.1021/es202417t

Yang, X., Jiang, C., Hsu-Kim, H., Badireddy, A.R., Dykstra, M., Wiesner, M., Hinton, D.E., Meyer, J.N. (2014). Silver nanoparticle behavior, uptake, and toxicity in *Caenorhabditis elegans*: effects of natural organic matter. Environ. Sci. Technol. 48, 3486–3495. https://doi.org/10.1021/es404444n

Yang, Y., Xu, G., Xu, S., Chen, S., Xu, A., Wu, L. (2018). Effect of ionic strength on bioaccumulation and toxicity of silver nanoparticles in *Caenorhabditis elegans*. Ecotoxicol. Environ. Saf. 165, 291–298. https://doi.org/10.1016/j.ecoenv.2018.09.008

Yang, Y., Xu, S., Xu, G., Liu, R., Xu, A., Chen, S., Wu, L. (2019). Effects of ionic strength on physicochemical properties and toxicity of silver nanoparticles. Sci. Total Environ. 647, 1088–1096. https://doi.org/10.1016/j.scitotenv.2018.08.064

Yang, Y.-F., Cheng, Y.-H., Liao, C.-M. (2017). Nematode-based biomarkers as critical risk indicators on assessing the impact of silver nanoparticles on soil ecosystems. Ecol. Indic. 75, 340–351. https://doi.org/10.1016/j.ecolind.2016.12.051

Zhang, W., Li, W., Li, J., Chang, X., Niu, S., Wu, T., Kong, L., Zhang, T., Tang, M., Xue, Y. (2021). Neurobehavior and neuron damage following prolonged exposure of silver nanoparticles with/without polyvinylpyrrolidone coating in Caenorhabditis elegans. J. Appl. Toxicol. JAT 41, 2055–2067. https://doi.org/10.1002/jat.4197
